# Exploring the cytotoxicity on human lung cancer cells and DNA binding stratagem of camptothecin functionalised silver nanoparticles through multi-spectroscopic, and calorimetric approach

**DOI:** 10.1038/s41598-023-34997-w

**Published:** 2023-06-03

**Authors:** Aparna Raj, Riju K. Thomas, L. Vidya, V. M. Aparna, S. Neelima, C. Sudarsanakumar

**Affiliations:** 1 School of Pure and Applied Physics, Mahatma Gandhi University, P.D Hills (P.O), Kottayam, Kerala 686 560 India; 2 Present Address: Bharata Mata College, Thrikkakara, Ernakulam, Kerala 682032 India

**Keywords:** Biophysics, Nanoscience and technology

## Abstract

The influence of nanoparticles inside the human body and their interactions with biological macromolecules need to be explored/studied prior to specific applications. The objective of this study is to find the potential of camptothecin functionalised silver nanoparticles (CMT-AgNPs) in biomedical applications. This article primarily investigates the binding stratagem of CMT-AgNPs with calf thymus DNA (ctDNA) through a series of spectroscopic and calorimetric methods and then analyses the anticancer activity and cytotoxicity of CMT-AgNPs. The nanoparticles were synthesized using a simple one pot method and characterized using UV–Visible, fourier transform infrared (FTIR) spectroscopy, X-ray diffraction and high-resolution transmission electron microscopy (HRTEM). The average size of CMT-AgNPs is 10 ± 2 nm. A group of experimental techniques such as UV–Visible spectrophotometry, fluorescence dye displacement assay, circular dichroism (CD) and viscosity analysis unravelled the typical groove binding mode of CMT-AgNPs with ctDNA. The CD measurement evidenced the minor conformational alterations of double helical structure of ctDNA in the presence of CMT-AgNPs. The information deduced from the isothermal titration calorimetry (ITC) experiment is that the binding was exothermic and spontaneous in nature. Moreover, all the thermodynamic binding parameters were extracted from the ITC data. The binding constants obtained from UV absorption experiments, fluorescence dye displacement studies and ITC were consistently in the order of 10^4^ Mol^−1^. All these results validated the formation of CMT-AgNPs–ctDNA complex and the results unambiguously confirm the typical groove binding mode of CMT-AgNPs. An exhaustive in vitro MTT assay by CMT-AgNPs and CMT against A549, HT29, HeLa and L929 cell lines revealed the capability of CMT-AgNPs as a potential anticancer agent.

## Introduction

Most recently, nanomaterials have arisen as crucial players in modern medicine. The unique properties like large surface to volume ratio, surface plasmon resonance, tuneable surface chemistry and high surface area make the nano-based materials as an excellent tool for biomedical applications^1–3^. These outstanding properties can be engineered or tuned to improve the bioavailability and biocompatibility for the development of new medical technologies. Among various nanomaterials, metal nanoparticles like gold and silver gained boundless research interests due to their unique physical, chemical and biological properties^4–6^. Especially, the silver nanoparticles (AgNPs) are known for its wide range of pharmacological properties like anti-bacterial, antifungal, anti-cancerous and anti-oxidant activities. Hence, the AgNPs of controlled morphology have been widely utilised in various bio related applications^5,7^. The synthesis of silver nanoparticles (NPs) through chemical and physical means suffers the setback of high cost and are toxic to the environment and the living organisms^8,9^. Hence the green approach for nanoparticle synthesis become more popular recently.

**Figure 1:**
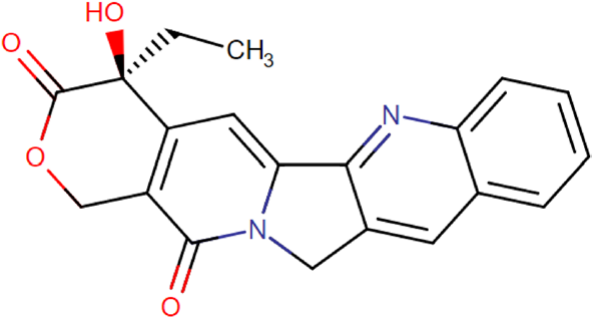
Structure of camptothecin.

Functionalisation of nanoparticles using plant extract, biomolecules, natural drugs, and microbes are helpful to fabricate eco-friendly and non-toxic nanoparticles of controlled size and shape^10,11^. An appropriate selection of capping agents will also offer high purity, stability, and better reproducibility. Organic compounds like flavonoids, alkaloids, and co-enzymes in plants can act as both reducing and stabilizing agents for nanoparticle synthesis, especially for AgNPs^12,13^. Alkaloids are a group of naturally occurring organic compounds that contain at least one nitrogen atom and are widely distributed in plants, fungi, bacteria, and animals. Camptothecin, chemically known as pyrano indolizino quinoline is one such alkaloid, isolated from the stem wood of the Chinese tree, *Camptotheca acuminata* (Fig. 1)^14^. Camptothecin (CMT) exhibits erratic anticancer activity in overture clinical trials, particularly against breast, lung, colon, and ovarian cancers. Besides being an anticancer agent, it has also shown anti-HIV activity^14–17^. Even though camptothecin is deeply investigated, its conjugate with AgNPs is not yet explored. While incorporating camptothecin with AgNPs, the properties could have enhanced so as the biomedical applications. However, before introducing a new material for biomedical applications, it is crucial to know its cytotoxicity and the interaction effects on biological macromolecules. In this context we synthesised the CMT-AgNPs and investigated its DNA interaction and anticancer potential on various human cancer cell lines and the cytotoxicity on L929 cells.

For binding studies, we selected calf thymus DNA (ctDNA) which is often used in the analysis on DNA binding anti-cancer agents^18–21^. The CMT-AgNPs can bind with ctDNA either through covalent or non-covalent bindings. The molecules that can bind non-covalently to DNA are of two types: Groove binders and intercalators^21,22^. Groove binders bind to the major or minor grooves of DNA via electrostatic interaction, hydrogen bonding, and van der Waals interactions^13^. Intercalating molecules will stack between progressive base pair of DNA through weak interactions like π-stacking^13^.

In the current work, we have synthesised CMT-AgNPs and systematically examined the interactions of CMT-AgNPs with ctDNA to demonstrate its binding mode and selectivity using calorimetric and spectroscopic techniques. The binding affinity of the nanoparticle is evaluated from the UV–Visible absorbance studies. The groove binding mode of CMT-AgNPs with ctDNA is disentangled by fluorescence dye displacement studies. The stability aspects and conformational changes of ctDNA in the presence of CMT-AgNPs were inferred from the viscosity measurements and Circular Dichroism (CD). The thermal kinetics associated with the interaction of CMT-AgNPs with ctDNA was studied from isothermal titration calorimetry (ITC). An exhaustive in vitro MTT assay on A549 cell lines were performed to find the concentration dependent anti-cancer activity. ROS and flow cytometry were also conducted to confirm the activity^23^. An in vitro MTT assay on HT29 and, HeLa cell lines were performed to confirm the anticancer potential of the sample. This investigation could also reveal the potential of CMT-AgNPs for the development of biomedical devices. For that cytotoxicity evaluation of CMT-AgNPs against L929 cell lines were conducted. The changes in absorption spectrum of ctDNA with addition of nanoparticles can be further studied to explore the feasibility of CMT-AgNPs as DNA sensor. The novelty of the current work includes the synthesis of CMT-AgNPs, characterizations and, DNA binding strategies of CMT-AgNPs. On top of that, anticancer activity and cytotoxicity studies of the camptothecin capped AgNPs are not presented so far. This article proposes camptothecin capped silver nanoparticles as a potential candidate for biomedical applications.

## Materials and methods

### Materials

Calf Thymus-DNA (sodium salt, Type l), Camptothecin with ≥ 90% (HPLC) purity, silver nitrate (AgNO_3_) ≥ 99%, Hoechst (Hst), Methyl green (MtG) and Phosphate buffer (pH 7.4) were purchased from Sigma-Aldrich. A549 Cells (Human lung cancer cells) and L929 cells were purchased from National Centre for Cell Sciences, Pune, INDIA and maintained in Dulbecco’s modified Eagles medium, DMEM (Sigma Aldrich, USA). The stock solution of ctDNA (200 μM) was made in Phosphate buffer (pH 7.4) and incubated for 24 h. All the other solvents/chemicals were of reagent grade and used without any further purification.

### Synthesis of camptothecin capped silver nanoparticles

A one-pot synthesis method was employed for the preparation of CMT functionalized silver nanoparticles using CMT as both reducing and capping agent^12,13^. 1 mM CMT dissolved in 0.01 mM NaOH (at 95 °C) and 1 mM AgNO_3_ were mixed together in 4:3 volume ratios with continuous stirring for 30 min at room temperature by keeping the solution at a stable pH of 7.4. The colour transformation of transparent solution to yellow revealed the formation of CMT-AgNPs. The resulting solution further centrifuged (3500 rpm, 1 h), washed multiple times with milli-Q-water for removing impurities effectively and finally dispersed in phosphate buffer solution of pH 7.4 for optical and calorimetric analysis.

### Instrumentation and experimental methods

#### X-ray diffractometry (XRD)

The crystalline natures of the CMT-AgNPs were investigated and the spectrum was recorded by PANalytical X-ray diffractometer (XRD) with Cu Kα radiation of wavelength λ = 1.5406 Å within the range of 2θ from 20° to 90°. The sample was prepared by drop-casting in a 1 cm × 1 cm glass slide and dried in the hot air oven^24^.

#### Attenuated total reflection-Fourier transform infrared spectroscopy (ATR-FTIR)

FTIR spectrum is taken to confirm the presence of CMT functionalization on the Ag nanoparticles by the structural vibrations acquired during the formation of CMT-AgNPs. The FTIR spectrum was taken using the Shimadzu model: IR-Prestige21, with a Diamond-crystal spectrometer from pike technologies over a range of 3900–600 cm^−1^. Spectra of camptothecin are taken in powder form whereas the CMT-AgNPs sample is prepared by drop-casting in a 1 cm × 1 cm glass slide dried in a hot air oven.

#### High resolution-transmission electron microscopy (HRTEM)

To directly examine the microstructural features of synthesized CMT-AgNPs, high-resolution transmission electron microscopy is used. HR-TEM images of the AgNPs are taken using JEOL-JEM-210 high resolution-transmission electron microscopy (HRTEM), with a LaB6 filament with an operating voltage of 200 kV equipped with EDX-OXFORD-8142. The sample preparation is by dropping two drops of the sample in a carbon-coated copper grid and letting it dry at room temperature^25^.

#### Atomic absorption spectroscopy (AAS)

The CMT-AgNPs concentration in the solution was estimated from atomic absorption spectroscopy (AAS) using a Perkin Elmer atomic photometer Model:—Pinnacle 900H. The concentration of silver in the solution obtained in the unit mg/L from AAS is converted into molarity using the equation^13^,

$${\text{Molarity }} = \, \left( {\left( {{\text{value in mg}}/{\text{L from AAS}}} \right) \, / \, \left( {\text{molecular weight of silver}} \right)} \right) \, \times { 1}000$$

#### UV–visible absorption spectroscopy

All absorption spectra measurements were taken using UV–Vis–NIR spectrophotometer (Agilent technologies). It is initially used to identify the prepared nanoparticle. UV–Visible spectroscopy is employed to evaluate the complex formation of CMT-AgNPs with ctDNA^26–29^. Absorbance spectrums of ctDNA in the presence of varying concentrations of CMT-AgNPs have accounted for across a range of 230 to 360 nm. Also, for obtaining a complete idea of the interaction of NPs and ctDNA, the absorbance spectrums of CMT-AgNPs were evaluated by increasing the ctDNA concentration. The binding affinity and Limit of Detection (LOD) were estimated from the Benesi-Hildebrand plot which is graphed according to the data from the UV–Vis spectrum^30^.

#### Competitive dye displacement assay—photoluminescence spectroscopy

Horiba-Fluoromax Spectrofluorometer is employed for all fluorescence measurements. The competitive dye displacement assay is done to unravel the binding mode of the CMT-AgNPs–ctDNA complex^31^. The DNA binding dyes Hst and MetG were used for the assay as it has an intense fluorescence intensity due to their known minor and major groove mode of binding with DNA respectively^32,33^. Thus, the nature of interaction or binding could be easily identified by tracking the modifications in fluorescence intensities of the dye–DNA complex by adding CMT-AgNPs.

#### DNA viscosity measurements

The Viscosity measurements were taken using an Ubbelohde-viscometer hung vertically at room temperature and a digital stopwatch is employed to adjust the flow time. The viscosity of the DNA–AgNPs complex is analysed to evaluate the mode of binding and confirm the result obtained from dye displacement studies^34,35^. The binding of DNA with small molecules may alter the chain length and this may result in a change of the viscosity of DNA. The intercalation mode of binding with DNA will cause an enlargement in the length of DNA along with a noticeable shift in the viscosity value of DNA due to the inclusion of molecules betwixt the base pairs. But the groove binding molecules will show a nominal or no characteristic change in the viscosity of the DNA^35–37^. The viscosity of the ctDNA is measured by the addition of varying concentrations of CMT-AgNPs in accordance with the ratio of [CMT-AgNPs]/[ctDNA]. To find the change of viscosity of DNA and the mode of binding, (η/η0)^1/3^ V/s ([CMT-AgNPs]/[ctDNA]) graph is plotted. η0 and η are the viscosity of ctDNA and ctDNA–CMT-AgNPs complex correspondingly.

#### Circular dichroism spectroscopy

The CD measurements of ctDNA and ctDNA-CMT-AgNPs complexes were examined over a range of 190 to 300 nm (far-UV range) at 25 °C using Jasco-J (715) Spectro-polarimeter. The path length is set to be 1 mm with a scan rate of 500 nm /min. The background spectrum of the buffer (Phosphate Buffer 7.4) was subtracted from the CD spectrum of the ctDNA and ctDNA–CMT–AgNPs complexes.

#### Isothermal calorimetry (ITC)

NanoITC from TA instruments, Germany is used to find the thermodynamics of the interaction of CMT-AgNPs with ctDNA^38–40^. About 10 μM solution of ctDNA and 50 μM solution of CMT-AgNPs were prepared in buffer (PBS-pH 7.4). This experiment is performed at 25 °C. 0.46μL of CMT-AgNPs solution was initially injected from the moving syringe into the sample cell and the volume of the next 19 injections was fixed as 2.46 μL. 20 injections were carried out and there is a time span of 300 s within two successive injections. The raw data was analysed by the NanoAnalyze software. The nature of the interaction and all the thermodynamic parameters except Gibbs free energy change (ΔG) were obtained directly from the software analysis. The change in Gibbs free energy (ΔG) can be estimated manually from the equation ΔG = ΔH − TΔS^39–41^.

#### Cytotoxicity analysis

L929 (adherent type of mouse fibroblast cell line), HeLa cells (human cervical carcinoma cells), HT29 (human colon adenocarcinoma cell lines) and A549 (human lung adenocarcinoma cell line derived from a primary lung tumour) cells were purchased from National Centre for Cell Sciences, Pune, INDIA and preserved in Dulbecco’s modified Eagle's medium, DMEM (Sigma Aldrich, USA). All the cell lines were cultured in 25 cm2 tissue culture flask with DMEM supplemented with 10% FBS, l-glutamine, sodium bicarbonate (Merck, Germany) and antibiotic solution containing: Penicillin (100 U/ml), Streptomycin (100 µg/ml), and Amphotericin B (2.5 µg/ml). Cultured cell lines were kept at 37ºC in a humidified 5% CO_2_ incubator (NBS Eppendorf, Germany).

Cells seeding in 96 well plate: two days old confluent monolayer of cells were trypsinized and the cells were suspended in 10% growth medium, 100 µl cell suspension (5 × 10^3^ cells/well) was seeded in 96 well tissue culture plate and incubated at 37 °C in a humidified 5% CO_2_ incubator.

Preparation of compound stock: the sample solution (CMT-AgNPs and CMT) was filtered through 0.22 µm Millipore syringe filter to ensure the sterility.

##### Cytotoxicity assay by MTT method

After 24 h the growth medium was removed, freshly prepared each compound in DMEM were five times serially diluted by two-fold dilution (100 µl, 50 µl, 25 µl, 12.5 µl, 6.25 µl in 500 µl of DMEM) and each concentration of 100 µl were added in triplicates to the respective wells and incubated at 37 °C in a humidified 5% CO_2_ incubator. Non treated control cells were also maintained. Fifteen mg of MTT (Sigma, M-5655) was reconstituted in 3 ml PBS until completely dissolved and sterilized by filter sterilization. After 24 h of incubation period, the sample content in wells were removed and 30 µl of reconstituted MTT solution was added to all test and cell control wells, the plate was gently shaken well, then incubated at 37 °C in a humidified 5% CO_2_ incubator for 4 h. After the incubation period, the supernatant was removed and 100 µl of MTT Solubilization Solution (Dimethyl sulphoxide, DMSO, Sigma Aldrich, USA) was added and the wells were mixed gently by pipetting up and down in order to solubilize the formazan crystals. The absorbance values were measured by using microplate reader at a wavelength of 540 nm^13,42^. LC50 values were calculated using ED50 PLUS V1.0 Software. The percentage of growth inhibition was calculated using the formula:

$$\mathrm{\% Viability }=\frac{Mean\, OD\, Samples\, \mathrm{X}\, 100}{Mean\, OD\, of\, control\, group}$$

*Detection of cell morphology* The viability of cells was evaluated by direct observation of cells. Entire plate was observed after 24 h of treatment in an inverted phase contrast tissue culture microscope (Olympus CKX41 with Optika Pro5 CCD camera) and the microscopic observations were recorded as images.

##### Detection of reactive oxygen species production

A549 cell line was cultured as per standard procedures described earlier. After attaining sufficient growth of A549 cells, the LC50 concentration of CMT-AgNPs was added and incubated for 24 h. untreated control cells were also maintained. Then the cells were washed with PBS and added with 50 µl of DCFDA (2’,7’-dichlorodihydro-fluoresciendiacetate), a florescent dye and incubated for 30 min to measure the ROS production. After incubation, excess dye was washed with PBS and fluorescence was imaged in a fluorescent microscope (Olympus CKX41 with Optika pro5 CCD camera) and fluorescence was measured using a fluorimeter at 470 nm excitation and emission at 635 nm (Qubit 3.0, Life technologies, USA) and expressed in arbitrary units^43^.

##### Apoptosis by flow cytometry

The FITC Annexin V/Dead Cell Apoptosis Kit with FITC annexin V and PI (propidium iodide) had used in apoptosis assay. A549 cell line was cultured as per standard procedures described earlier. After attaining sufficient confluency, LC50 concentration of sample added and incubated for 24 h. Untreated control wells were also maintained. The cell sample was transferred to a 12 × 75 mm polystyrene tube. The minimum recommended number of cells for fixation in a tube is 1 × 10^6^ cells. The samples were then centrifuged at 3000 rpm for 5 min. The supernatant was removed without disturbing the pellet. After centrifugation, the cell pellet forms either a visible pellet or a white film on the bottom of the tube. To the tubes added 100μL of the Muse™ Annexin V & Dead Cell Reagent to each tube. The tubes were mixed thoroughly by pipetting up and down or vortex at a medium speed for 3 to 5 s followed by incubation for 20 min at room temperature in the dark. The cells were analysed in a flow cytometer and then analysed using Muse flow cytometry software. The cells were gated against untreated control cells and analysed for apoptosis using Muse FCS 3.0 software^23,44^.

## Results and discussions

### Biophysical characterization of CMT-AgNPs

#### UV–visible absorption, FTIR and XRD analysis.

**Figure 2:**
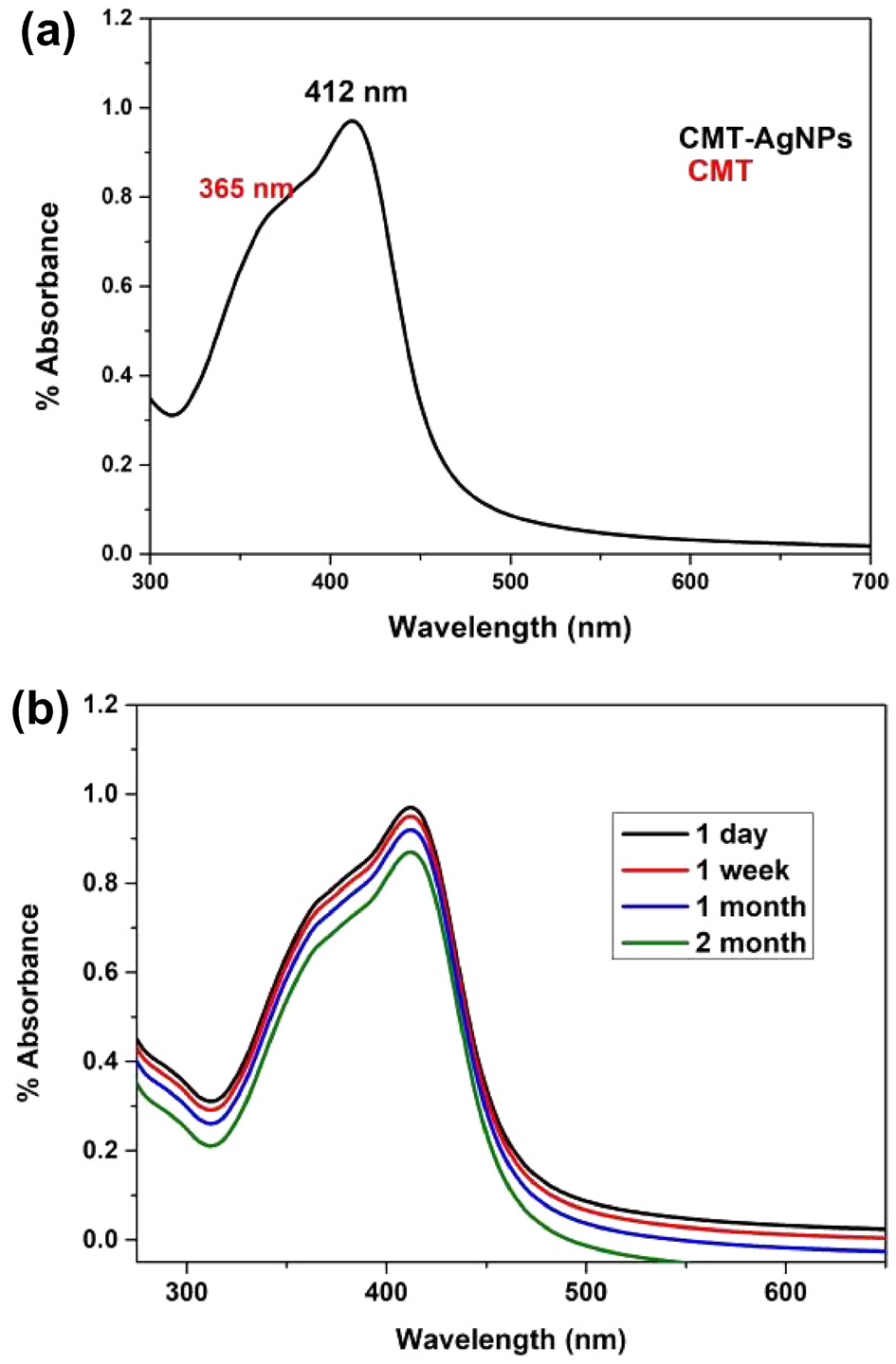
(**a**) UV–visible spectrum of CMT-AgNPs (yellow solution) showing the maximum LSPR intensity at 412 nm, (**b**) changes in the LSPR peak showing the stability of nanoparticles for 2 months.

The optical characteristics of the synthesized CMT-AgNPs were studied from its UV–Visible absorption spectrum. The absorption spectrum of camptothecin functionalized silver nanoparticles (CMT-AgNPs) is shown in Fig. 2a. The spectrum shows an intense absorption peak at 412 nm with a shoulder peak at 365 nm. The peak at 412 nm corresponds to silver nanoparticles' localised surface plasmon resonance (LSPR)^45^. This confirms the formation of CMT-AgNPs. The shoulder peak around 365 nm represents the UV peak of camptothecin^17^. To check the stability of nanoparticles, the LSPR spectrum was taken in different intervals for 2 months at room temperature. It was corroborated that the prepared CMT-AgNPs were highly stable at room temperature, devoid of intensity variation in the LSPR peak (Fig. 2b).

**Figure 3:**
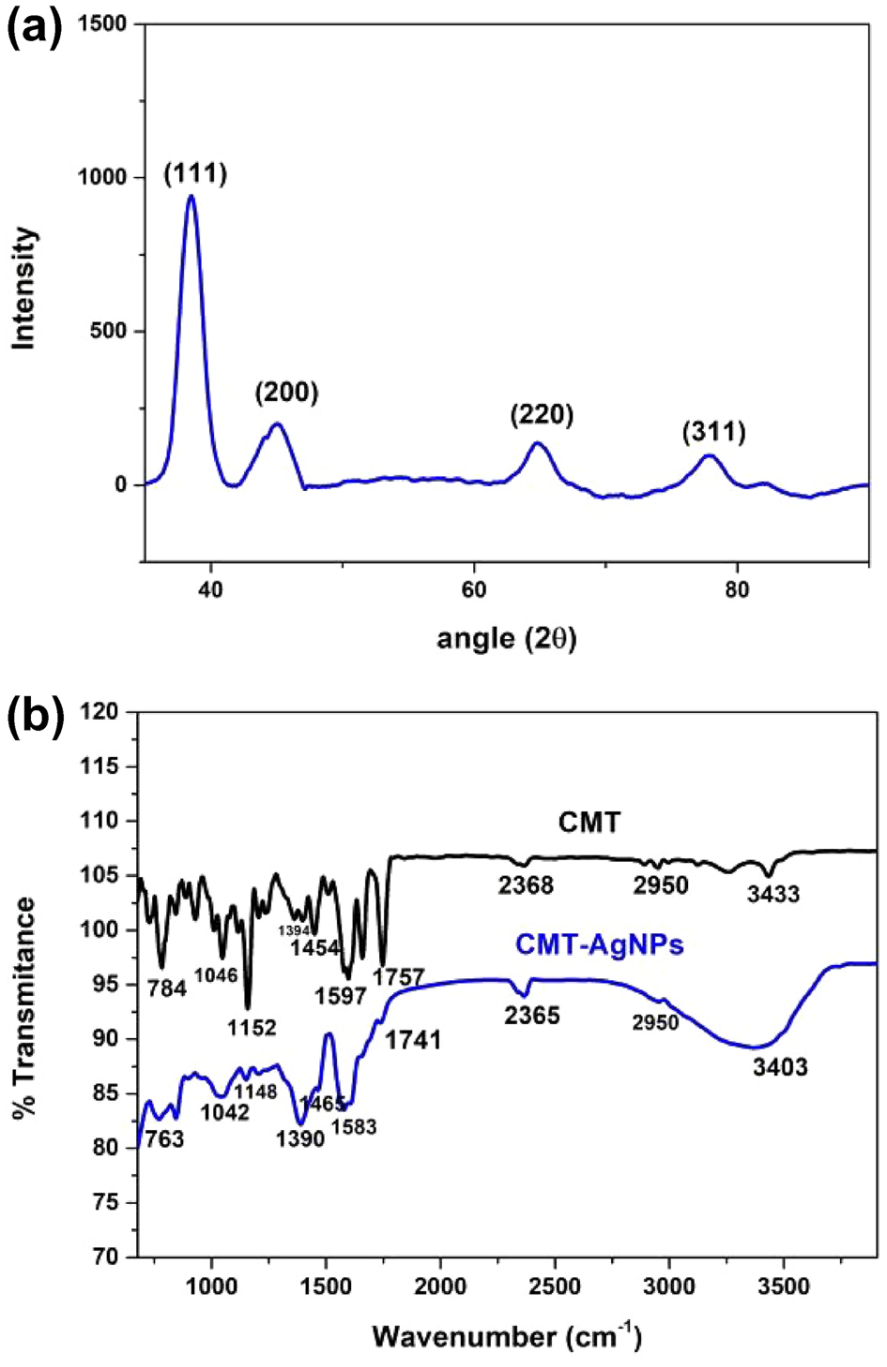
(**a**) XRD pattern of CMT-AgNPs. The planes are marked as reported in JCPDS card no: 04-0783. (**b**) FTIR spectrums of CMT and CMT-AgNPs.

The XRD spectrum recorded with 2θ value ranges from 20° to 90° are shown in Fig. 3a. The four dominant diffraction peaks in the spectrum were identified from JCPDS card no: 04-0783 and the peaks are recognized as (111), (200), (220), and (311) planes corresponding to face-centred cubic (fcc) structure of AgNPs^46,47^.

The FTIR spectra of camptothecin and CMT capped AgNPs are shown in Fig. 3b. The IR peak observed at 3433 cm^−1^ attributes to -OH stretching band, 2950 cm^−1^ corresponds to –CH and –CH_2_ bands and 1152 cm^−1^ ascribed to C–O stretching vibrations. Peaks at 1757 cm^−1^ and 1658 cm^−1^ refers to ester bond and C=O stretching vibration respectively, the peak at 1394 cm^−1^ is in concurrence with the C=N stretching band, the peak at 1454 cm^−1^ is in agreement with the –CH_3_ bands and the medium intense peak at 1597 cm^−1^ confirms the C=C (in ring) stretching vibration of the aromatic rings of the CMT^48^. But in the FTIR spectrum of CMT-AgNPs, the characteristic IR peaks of CMT corresponding to –OH (3433 cm^−1^–3403 cm^−1^) C–O (1152 cm^−1^–1042 cm^−1^) stretching vibrations and ester bond (1757 cm^−1^–1741 cm^−1^) depicted a downward shift together with an intensity decrease. This shift in peaks may mainly due to the binding of Ag^+^ ions to the hydroxyl group or to the ester group present in CMT. FTIR spectral analysis suggests the successful encapsulation of CMT on the surface of AgNPs^48,49^.

#### Particle size & morphology analysis by TEM

**Figure 4:**
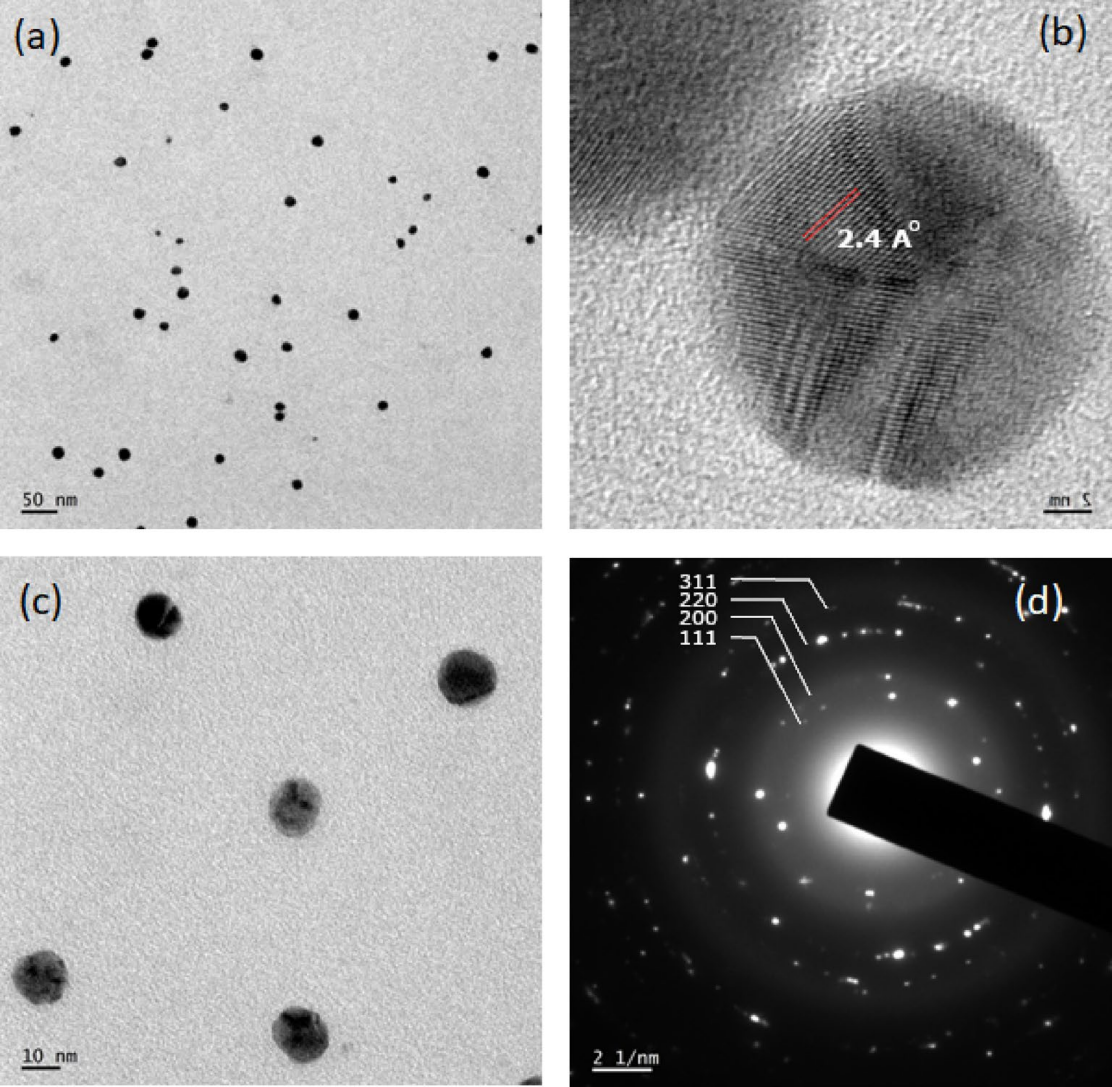
(**a,b**) TEM, (**c**) HRTEM, (**d**) SAED pattern of CMT-AgNPs.

The details about the particle size and the morphology of CMT-AgNPs were revealed from TEM analysis (Fig. 4). TEM images are shown in Fig. 4a, c. Almost all the particles have a spherical shape with an average particle size of 10 ± 2 nm. HRTEM image confirms the lattice planes and the “d” spacing value is calculated to be 2.4 Å from Fig. 4b. Figure 4d is representing selected area electron diffraction (SAED) pattern which shows the well-defined diffraction rings corresponding to the planes (111), (200), (220), and (311). The consistency of HRTEM and SAED pattern with the XRD spectrum substantiate the fcc crystalline structure of CMT-AgNPs.

### Investigating the interaction of CMT-AgNPs with ctDNA

We have successfully synthesized the CMT-AgNPs with good stability. The concentration of the CMT-AgNPs in the stock solution was calculated using the Atomic Absorption Spectroscopy (AAS). The concentration of silver in the solution obtained as 17 mg/L from AAS, it is converted in to molar concentration using the equation^13^,

$${\text{Molarity }} = \, \left( {\left( 17 \, {{\text{mg/L}}} \right) \, / \, \left( {\text{molecular weight of silver}} \right)} \right) \, \times {1}000$$

$${\text{Molar concentration of CMT - AgNPs }} = \, \left( 17/ 107.87 \right) \, \times {1}000 \, = 157.59\;{\upmu {\rm M}}$$

The concentration of stock solution of ctDNA was calculated using UV–Visible absorbance spectroscopy. Absorbance intensity at 260 nm (A_260_) and 280 nm (A_280_) are noted from the spectrum of ctDNA. Then the concentration and stability of ctDNA is calculated using Beer lambert's formula, A_260_/A_280_ ratio and the average excitation coefficient (E_260_ = 6600 Lmol^−1^ cm^−1^) at 260 nm.

To unveil the effect of these camptothecin-encapsulated silver nanoparticles on DNA, the interaction of H-AgNPs with ctDNA was evaluated with the help of spectroscopic and thermodynamic studies.

#### UV–visible absorption studies of CMT-AgNPs with ctDNA

**Figure 5:**
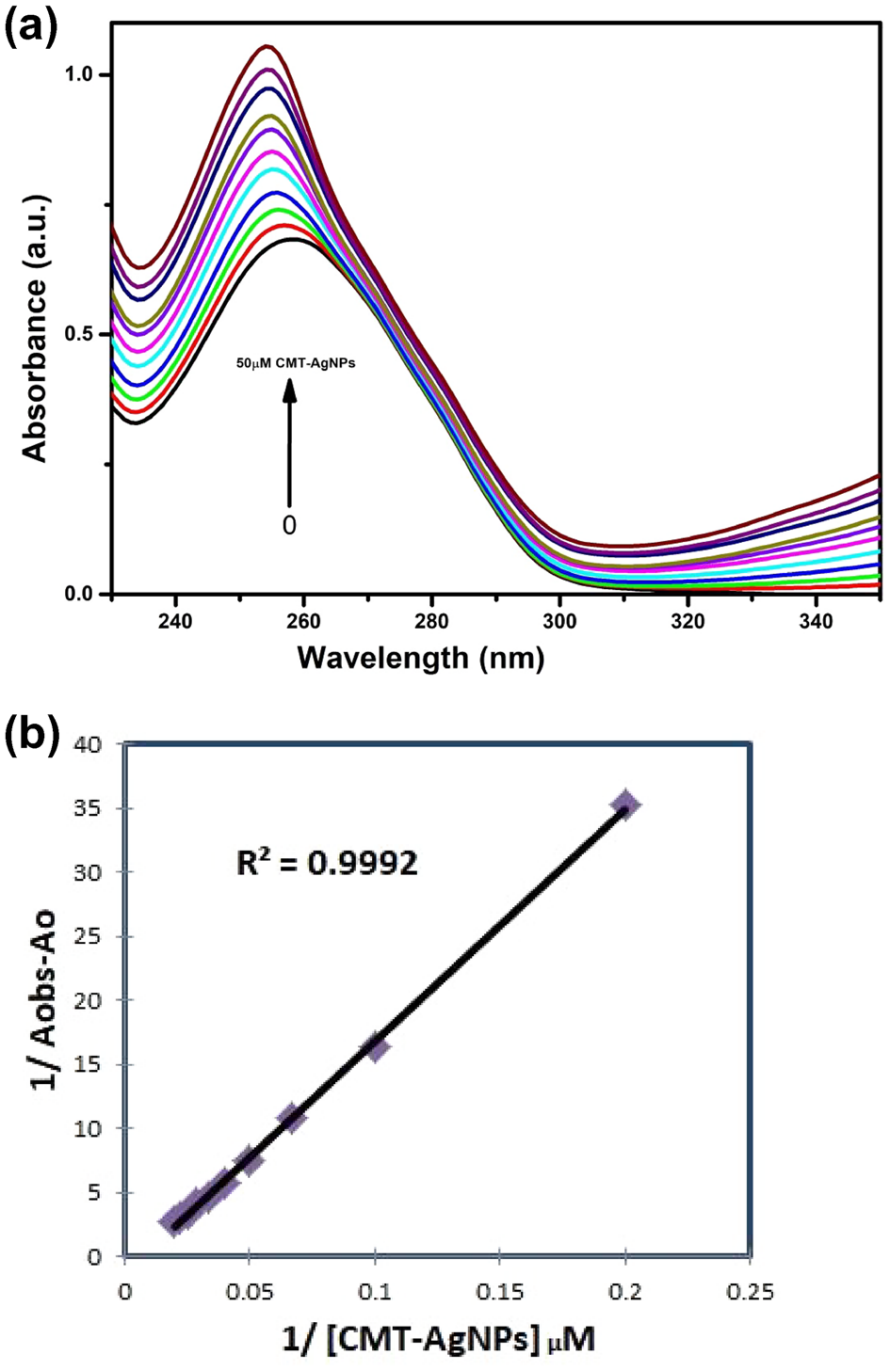
(**a**) UV–Vis absorption spectra of ctDNA (110 μM) in PBS (pH 7.4) with progressive concentrations of CMT-AgNPs (0 to 50 × 10^-6^ M), (**b**) Benesi–Hildebrand plot showing the correlation of 1/(A_obs_ – A_o_) and inverse of concentration of [CMT-AgNPs].

To analyse the binding affinity of the ctDNA towards CMT-AgNPs, UV–Visible absorption studies were carried out by taking the spectra of ctDNA of fixed concentration (110 μM) with increasing concentrations of CMT-AgNPs (0 to 50 × 10^-6^ M). Depending on the nature of interaction of the macromolecules, DNA will exhibit hyperchromic or hypochromic effects^50–53^. Hyperchromicity depicts the rise in material’s capacity of absorbing light while hypochromicity is to decrease. In the current study, we had observed a hyperchromic effect, i.e., a gradual rise in the absorption maxima of ctDNA while adding CMT-AgNPs of varying concentrations. Hyperchromic effect at 258 nm together with a bathochromic shift of 4 nm is illustrated in the Fig. 5a. However here a very slight characteristic shift in the absorption wavelength & intensity indicates a minimal structural alteration on ctDNA upon its direct complex formation with CMT-AgNPs^52,53^.

The Benesi–Hildebrand equation is employed to evaluate the binding energy constant **(K**_**σ**_**)** for the molecular complex formed of DNA and nanoparticle^54,55^.

The Benesi–Hildebrand equation is,

1 $${\mathbf{A}}_{{{\mathbf{obs}}}} = \, \left( {{\mathbf{1}} - {{\varvec{\upsigma}}}} \right) \, {\mathbf{M}}_{{\mathbf{0}}} {\mathbf{\upepsilon}}_{{{\mathbf{dna}}}} {\mathbf{l}} \, + \, {\mathbf{\sigma \upepsilon}} \, {\mathbf{l}}$$

where **A**_**obs**_ is the observed absorbance of ctDNA in the presence of different concentrations of CMT-AgNPs, **σ** is the degree of association between ctDNA and CMT-AgNPs, **ε**_**dna**_ and **ε** are the molar extinction coefficients of ctDNA and ctDNA–CMT-AgNPs complex respectively, **M**_**0**_ is the initial concentration of ctDNA and **l** is the optical path length.

The above equation can be expressed as

2 $${\mathbf{A}}_{{{\mathbf{obs}}}} = \, \left( {{\mathbf{1}} - {{\varvec{\upsigma}}}} \right) \, {\mathbf{A}}_{{\mathbf{o}}} + \, {\mathbf{\sigma A}}_{{\mathbf{c}}}$$

Where, **A**_**obs**_ and **A**_**o**_ are the absorbance (at absorption maximum) of ctDNA and ctDNA-nanoparticle complex respectively.

In a relatively high nanoparticle concentration, **σ** can be equated to **(K**_**σ**_**[NPs])/(1 + K**_**σ**_** [NPs])** and the above equation can be expressed as,

3 $${\mathbf{1}}/\left( {{\mathbf{A}}_{{{\mathbf{obs}}}} - {\mathbf{A}}_{{\mathbf{o}}} } \right) \, = \, {\mathbf{1}}/\left( {{\mathbf{A}}_{{\mathbf{c}}} - {\mathbf{A}}_{{\mathbf{o}}} } \right) \, + \, {\mathbf{1}}/ \, \left( {{\mathbf{K}}_{{{\varvec{\upsigma}}}} \left( {{\mathbf{A}}_{{\mathbf{c}}} - {\mathbf{A}}_{{\mathbf{o}}} } \right) \, \left[ {{\mathbf{NPs}}} \right]} \right)$$

Figure 5b is the plot representing the relationship between 1/(A_obs_ − A_o_) and 1/[CMT-AgNPs]. Slope of the plot is the value of 1/(K_σ_(A_c_ − A_o_) while intercept is 1/(A_c_ − A_o_). From these values the binding constant can be calculated using the Eq. (3) and the value is found as 0.68 × 10^4^ M^−1^. From the UV–Vis absorption studies it can be concluded that CMT-AgNPs and ctDNA exhibit a solid complex formation.

**Figure 6:**
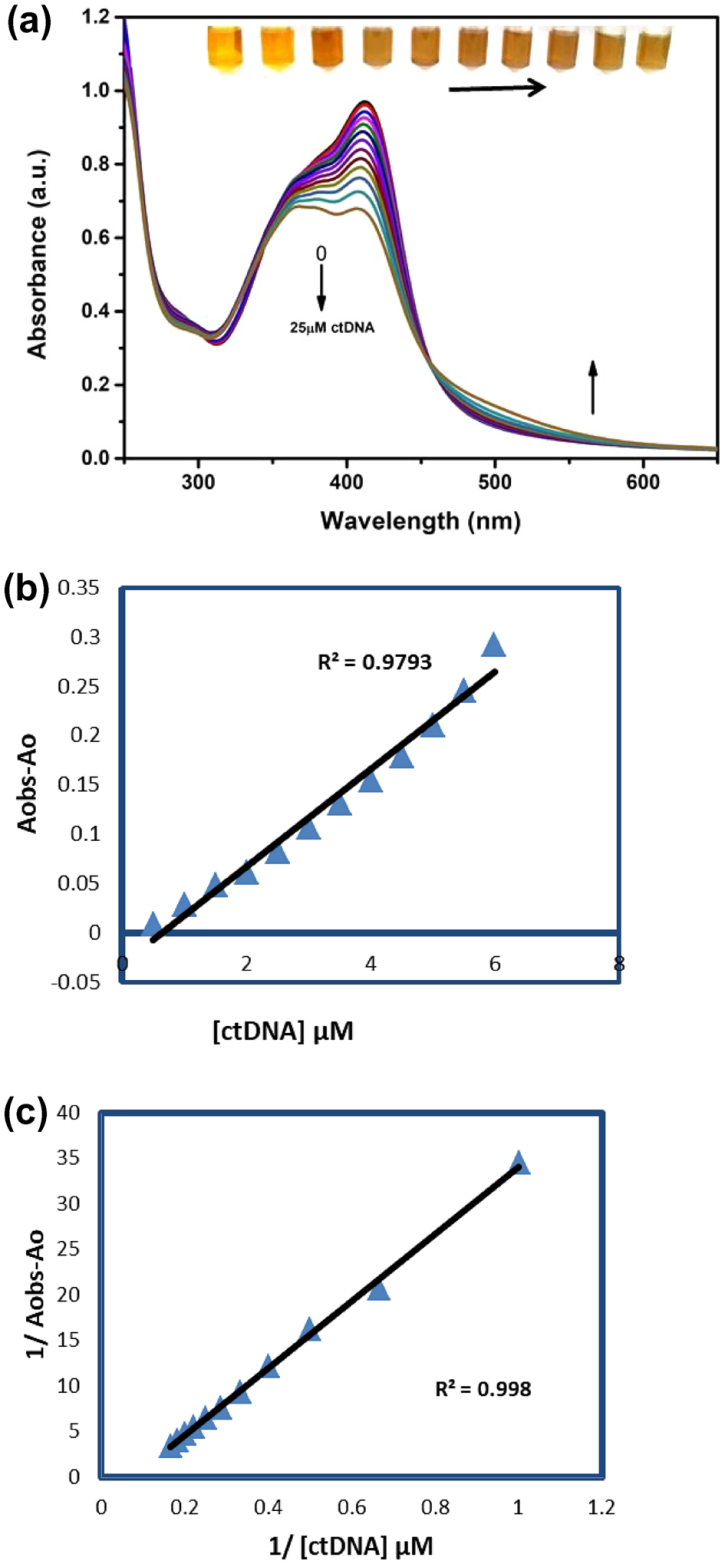
(**a**) Absorption spectra of CMT-AgNPs (LSPR) in the presence of different concentrations of ctDNA (0-25 μM), (**b**) linear plot of change in absorbance of CMT-AgNPs with ctDNA concentration (0–25 μM), (**c**) plot of inverse of A_obs_–A_o_ versus inverse of ctDNA concentrations (Benesi–Hildebrand plot).

To obtain a complete idea about the interaction between NPs and ctDNA, the LSPR spectrum of CMT-AgNPs was taken by varying the concentration of ctDNA from 0 to 25 μM. The decrease in the intensity of LSPR peak at 412 nm without any characteristic shift confirms that there are no significant changes in the di-electric microenvironment of CMT-AgNPs due to their bonding with ctDNA. The inset picture in Fig. 6a represents the gradual colour change of CMT-AgNPs solution from yellow to light brown colour with the addition of increasing concentrations of ctDNA.

Figure 6b represents the plot of A_obs_–A_o_ versus ctDNA concentration added to CMT-AgNPs. Limit of detection (LOD) can be found using the Eq. (3) σ/K, where K represents the slope of the graph and σ represents the standard deviation. The interaction potency of CMT-AgNPs and ctDNA was measured from the Benesi–Hildebrand plot drawn using LSPR data (Fig. 6c). The binding constant and the limit of detection (LOD) calculated from the plots were 1.8 × 10^4^ M^−1^ and 2.7 μM respectively. The above results suggested the DNA detection efficiency of CMT-AgNPs by means of its binding mechanism.

#### Fluorescence quenching studies of ctDNA using competitive dye displacement assay

**Figure 7:**
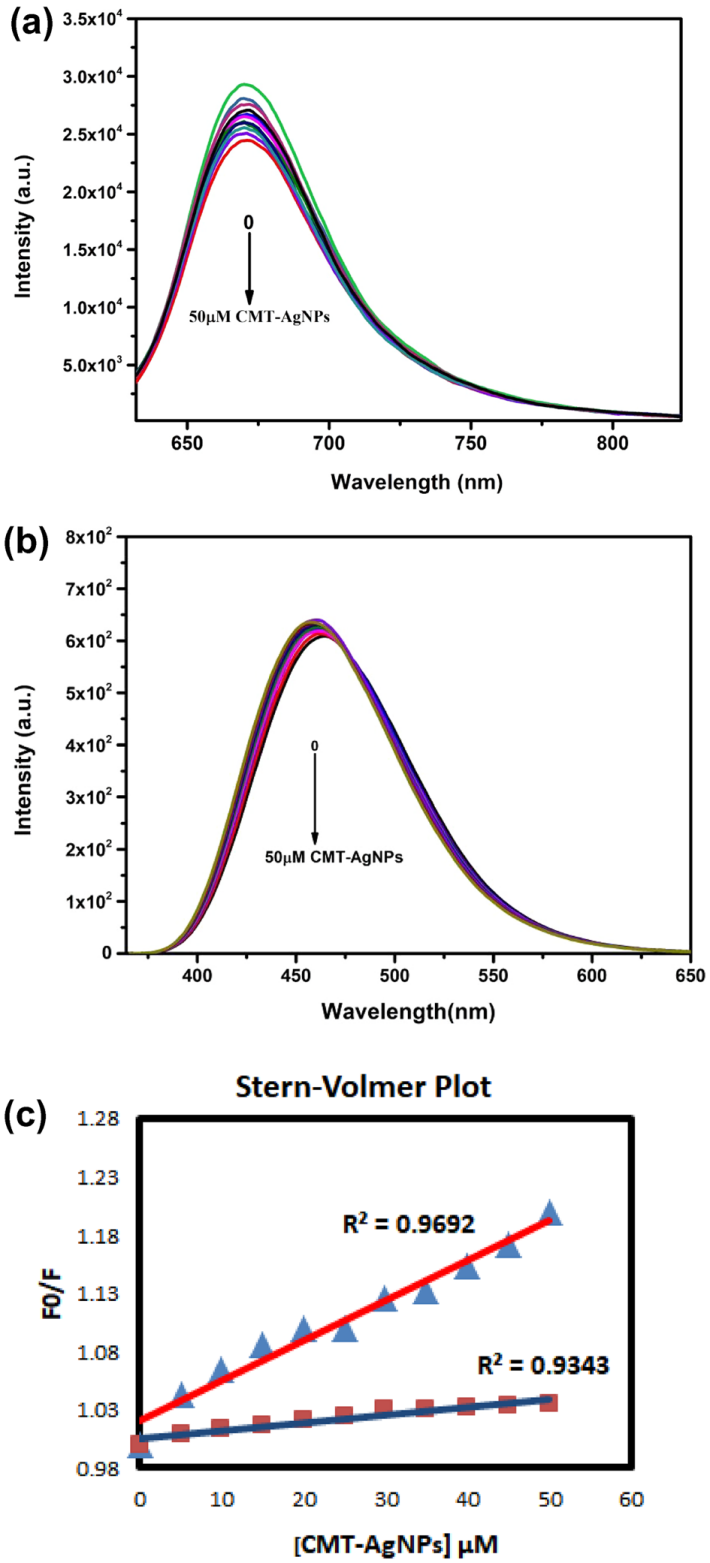
Fluorescence emission spectra of the (**a**) MetG–ctDNA (ctDNA-75 μM, MetG-5 μM), (**b**) Hst–ctDNA complex (ctDNA-75 μM, Hst-5 μM)) in the presence of CMT-AgNPs (0–50 μM) and (**c**) The Stern–Volmer plot of ctDNA–MetG (red line) and Hst–ctDNA (blue line) complex with the varying concentration of CMT-AgNPs from 0 to 50 μM.

A dye–DNA complex can be excited by a specific wavelength and will simultaneously emit light of a characteristic wavelength depending on the dye. With the help of these dyes, it is easy to identify the binding mode of nanoparticles to the DNA^56^. In the present study, we had used Hoechst (Hst), a minor groove binder and Methyl green (MetG), a major groove binder dyes to reveal the minor/major groove binding mode of CMT-AgNPs to ctDNA. The complex formed of Hst and ctDNA shows an intense fluorescence spectrum with a maximum emission peak around 461 nm when excited at 350 nm^53,57^. The MetG–ctDNA complex system shows the maximum emission intensity peak around 663 nm when excited at 677 nm^58^. The subsequent addition of CMT-AgNPs (0–50 μM) to the MetG–ctDNA complex resulted in significant quenching of emission intensity suggesting the replacement of MetG dye with CMT-AgNPs from the major groove of ctDNA (Fig. 7a). These results disclosed that CMT-AgNPs competed with MetG for binding in the major groove of ctDNA and succeeded by the displacement of MetG^59^. Figure 7b is the Emission spectrum of the Hst–ctDNA system with addition of different concentrations of CMT-AgNPs from 0 to 50 μM. The negligible change in the intensity upon the addition of CMT-AgNPs evidenced that the CMT-AgNPs did not replace Hst from the Hst–ctDNA complex. This rule out the probability of minor groove binding of CMT-Ag nanoparticles with ctDNA since Hoechst dye is a strong minor groove binder. From the above results, the major groove binding pattern of CMT-AgNPs to ctDNA is confirmed.

The quenching of ctDNA by CMT-AgNPs can be analysed by Stern–Volmer plot.

4 $${\mathbf{F}}_{{\mathbf{o}}} /{\mathbf{F}} \, = \, {\mathbf{1}} \, + \, {\mathbf{S}}_{{\text{v}}} \left[ {{\mathbf{CMT}} - {\mathbf{AgNPs}}} \right]$$

Where, **F**_**o**_ is the intensity maximum of ctDNA-dye system while **F** represents the same in the presence of CMT-AgNPs. **S**_v_ is the Stern–Volmer quenching constant and [CMT-AgNPs] is the concentration of CMT-AgNPs. Figure 7c is the Stern–Volmer (S–V) plot of dye–ctDNA–[CMT-AgNPs] complex. The slope of the S–V plot represents the value of **S**_v_. The Stern–Volmer quenching constant for MetG and Hst displacement was found to be 3.5 × 10^3^ M^−1^ and 7 × 10^2^ M^−1^ respectively. The MetG–ctDNA system has higher **S**_v_ value in the presence of CMT-AgNPs when compared to Hst–ctDNA. Thus, it was evident from this dye displacement assay that CMT-AgNPs bind in the major groove of ctDNA excluding the possibility of minor groove mode of binding with ctDNA.

#### DNA viscosity measurements

**Figure 8:**
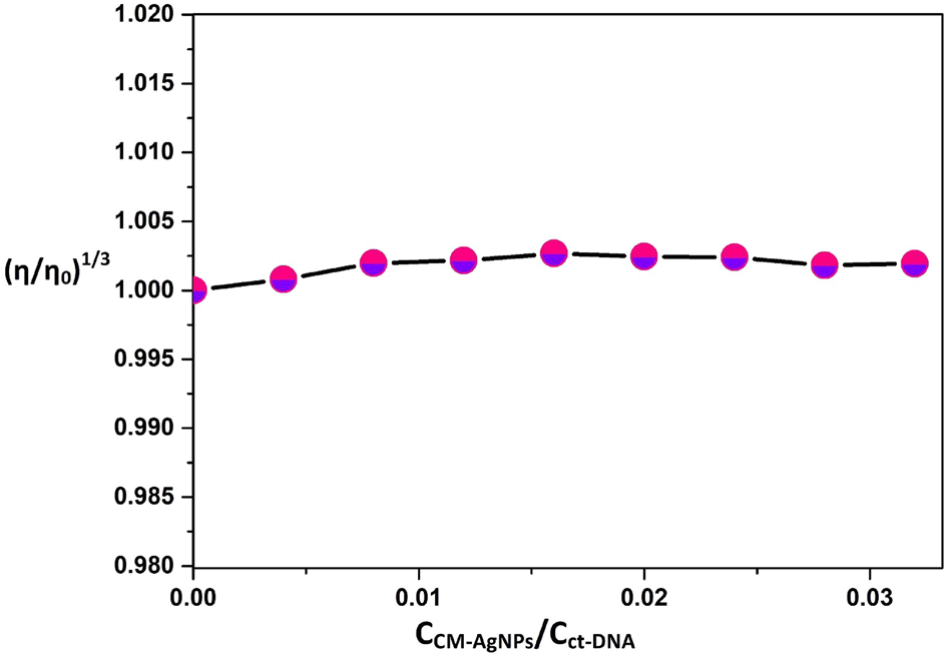
Viscosity measurement graph of ctDNA with the increasing concentration of CMT-AgNPs. The ctDNA concentration (50 μM) was kept constant while increasing the concentration of CMT-AgNPs from 0 to 20 μM.

Viscosity of the ctDNA is measured by the addition of varying concentration of CMT-AgNPs in accordance with the ratio of [CMT-AgNPs]/[ctDNA]. To find the change of viscosity of DNA and the mode of binding, (η/η0)^1/3^ V/s [CMT-AgNPs]/[ctDNA] graph is plotted. η0 and η are the viscosity of ctDNA and ctDNA–CMT-AgNPs complexes respectively. The viscosity measurement graph shows a good linearity (Fig. 8). From the linear graph it was confirmed that CMT-AgNPs put forth no apparent effect on the viscosity of ctDNA solution. This excludes the probability of intercalation binding and corroborated the groove binding eventuality of CMT-AgNPs to the ctDNA^38^. These findings are consistent with the conclusions obtained from dye displacement studies.

#### Isothermal titration calorimetric analysis (ITC)

**Figure 9:**
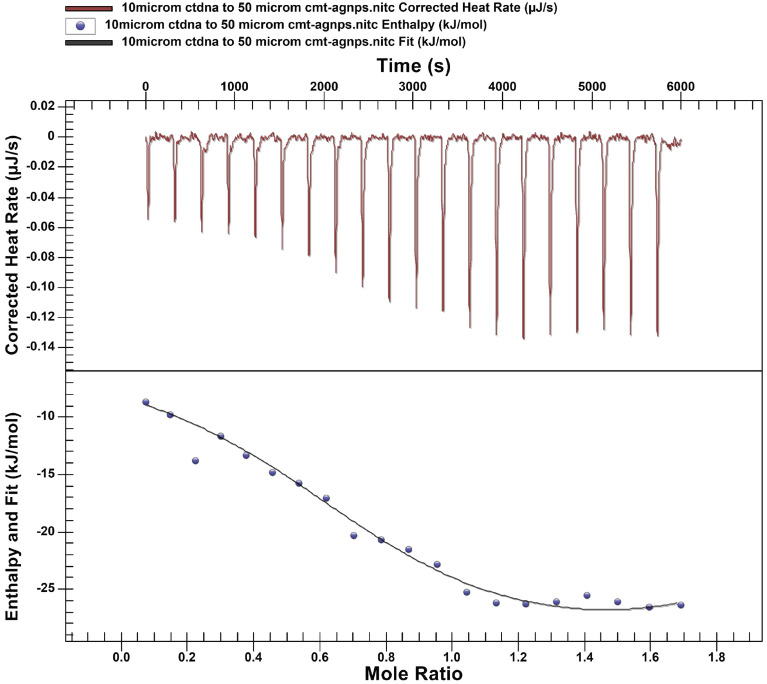
ITC Profile showing the interaction of CMT-AgNPs with ctDNA at 298 K.

Isothermal calorimetric analysis is used to find the nature of reaction and all the thermodynamic variables like the Gibbs free energy, entropy, enthalpy of a reaction directly from a single titration experiment. The Nano ITC is designed for low volume titrations and in turn will give maximum sensitivity and flexibility for the study of bio molecular binding. Here ITC was effectively used to disclose the thermodynamics associated with the interaction of CMT-AgNPs with ctDNA^10,18,19^. The interaction nature is found to be exothermic. The row ITC data collected from the experiment were fitted by the ‘multiple binding site model’. The data was fitted with binding stoichiometry n = 2. The overlay graph (Fig. 9) includes two parts, the upper part is the raw ITC curve due to the 20 injections of CMT-AgNPs to the ctDNA in the cell and the lower part is the fitted plot using multiple binding site models.

The values of ΔH, ΔS, Gibbs free energy and affinity constant (K_a_) were calculated at 298 K and listed in Table 1. Binding forces acted can be identified using the sign and magnitude of the obtained thermodynamic variables^10^. For hydrogen bonding ΔG, ΔH and ΔS are –ve but for hydrophobic interactions ΔH and ΔS are + ve and ΔG is –ve. In the case of electrostatic interactions ΔS is + ve and ΔG is −ve but ΔH can be either + ve or −ve. According to the Table 1 data, complex formed by CMT-AgNPs and ctDNA were stabilised in two binding sites N_1_ and N_2_. The N_1_ binding site is stabilized due to the hydrogen bonding and Van der Waals, while the N_2_ site is by electrostatic interactions^10,18,19^. The value of binding constant or association constant of CMT-AgNPs with ctDNA in two binding sites are 8.37 ± 0.65 × 10^5^ M^−1^ and 2.32 ± 0.59 × 10^4^ M^−1^, which again proved the existence of strong binding of CMT-AgNPs with ctDNA. The present interaction is exothermic and enthalpy driven with slight entropic contribution^60^. This confirms the groove binding mode of CMT-AgNPs to ctDNA^60^.

**Table 1:** Thermodynamic parameters obtained from ITC profile.

Binding sites	ΔH ( kJ/mol)	ΔS ( J/mol K)	K_a_ (M^−1^)	ΔG (kJ/mol)
N_1_	−2.14 ± 0.49	116.36 ± 13.97	8.37 ± 0.65 × 10^5^	−39.82 ± .35
N_2_	−138.16 ± 67.6	−490.76 ± 86.05	2.32 ± 0.59 × 10^4^	−8.25 ± 0.66

#### Conformational aspects of binding by CD spectroscopy

The circular dichroism spectroscopic studies were conducted in order to find the conformational alterations of ctDNA due to the binding of CMT-AgNPs. The basic CD spectrum of a bare DNA has a + ve band and a − ve band at 275 nm and 245 nm respectively. The right-handed helicity of DNA is designated by the − ve band at 245 nm while the stacking interaction (π–π) between the DNA base pairs is attributed to + ve band at 275 nm^61–65^. Any non-covalent ligand interactions may induce structural changes on DNA and therefore the characteristic nature of the CD spectrum will be altered^61,64^. During intercalation there will be significant alterations in both the bands, while in electrostatic or groove bindings interactions DNA suffers any variations^64^.

**Figure 10:**
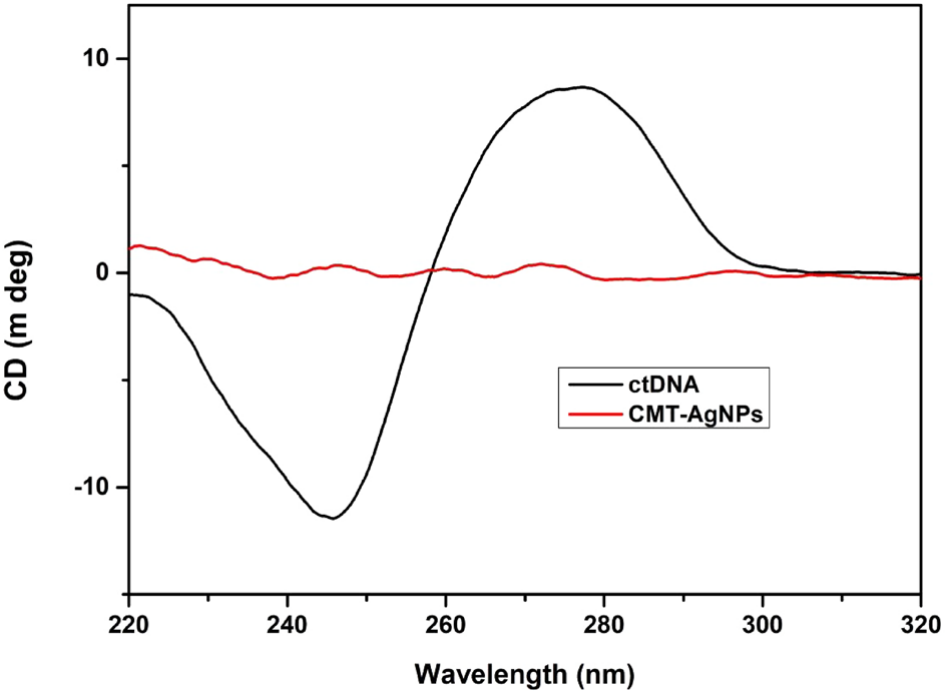
CD spectra of ctDNA and CMT-AgNPs.

**Figure 11:**
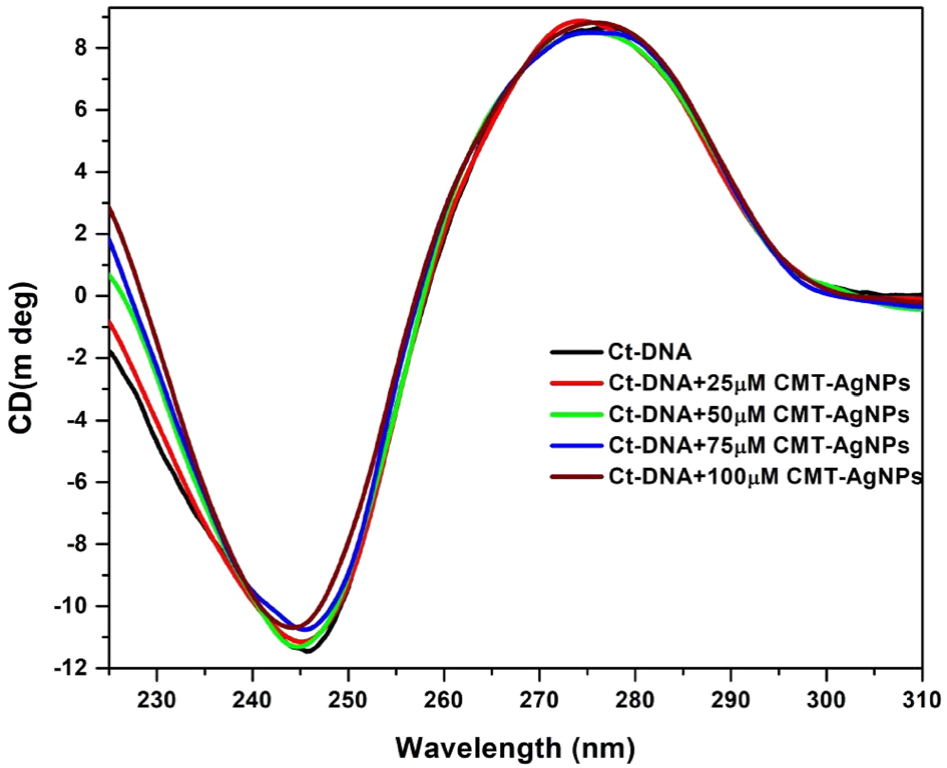
CD spectra of ctDNA (in PBS pH 7.4) with increasing concentration of CMT-AgNPs at room temperature.

The characteristic CD spectra of ctDNA and CMT-AgNPs are shown in Fig. 10. It is inferred from the figure that CMT-AgNPs did not show any intrinsic optical activity. Figure 11 represents the CD spectra of ctDNA alone and ctDNA with the addition of increasing concentration of CMT-AgNPs. These spectra show that the negative band of ctDNA exhibits insignificant changes with varying concentrations of CMT-AgNPs (0–20 μM) while no changes in the positive band indicate no conformational variations to the base stacking^61–65^. Here we have observed tiny changes in the band at 245 nm in accordance with the slight deformity in the helical structure of ctDNA, which is due to the typical groove binding mode of CMT-AgNPs^13,61,64^. This confirms the groove binding fashion of CMT-NPs since it is already known that the groove binders do not affect the base stacking and may lead to the perturbation of DNA duplex helicity^13^. The CD spectroscopic analysis is evenly matched with the dye displacement analysis and viscosity studies.

### Anticancer activity of CMT-AgNPs

Camptothecin (CMT) and its derivatives are natural drugs that are well-known for the anticancer property on lung cancer cells^66–69^. Since we incorporated camptothecin as a functionalising agent, the anticancer activity analysis of CMT-AgNPs is also important. In the present study, we have investigated the anticancer activity of CMT-AgNPs against A549 cells. An exhaustive in vitro MTT assay on A549 cells (Human lung cancer cells) were done in a dose dependent manner. To compare the anticancer potential of CMT-AgNPs, an MTT assay was conducted on CMT against A549 cells. The morphological changes due to CMT-AgNPs and CMT in the lung cancer cells were visualised through the optical microscope^70^. Non treated cells were used as a negative control and doxorubicin is used as a positive control for MTT assay^71,72^. ROS detection and flow cytometry is conducted followed by MTT assay^43^. The analysis on cytotoxicity of nanoparticles is important for their safe application in biosystems. To explore the possibility of CMT-AgNPs for biomedical applications, we have followed ISO 10993-5:2009 and conducted an in vitro MTT assay on L929 cells^73–75^. To ensure the anticancer property of nanoparticles, two other cancer cells were also used to analyse CMT-AgNPs. An MTT assay was conducted on cervical cancer and colon cancer cell lines to further confirm the anticancer activity. Since the CMT-AgNPs are monodispersed in water, concentration of sample is taken as µl/ml for all the cytotoxicity experiments. The quantity of camptothecin is taken for toxicity analysis is as the same amount used for the synthesis of CMT-AgNPs.

#### Anticancer activity on human lung cancer cell lines

##### MTT assay

**Figure 12:**
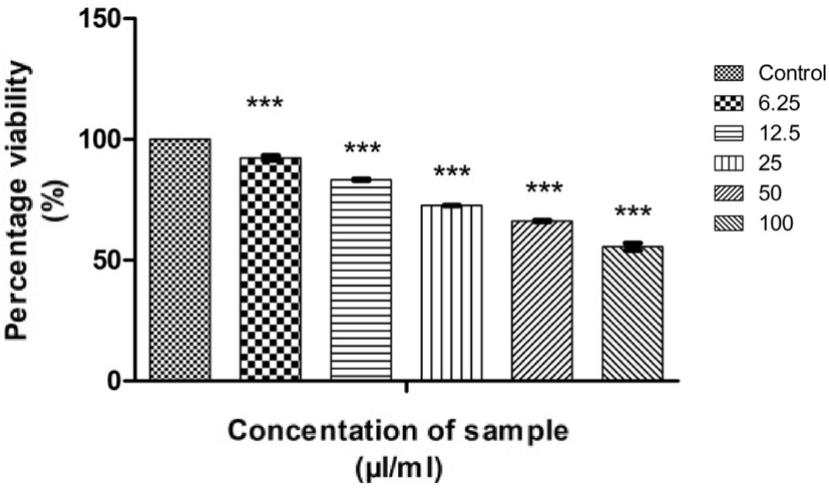
Graphical representation depicting the cytotoxic effect of CMT-AgNPs on A549 cell lines by MTT assay. Data was shown as the mean ± standard deviation (experiments = 3), ***p < 0.001 compared to untreated cells.

**Figure 13:**
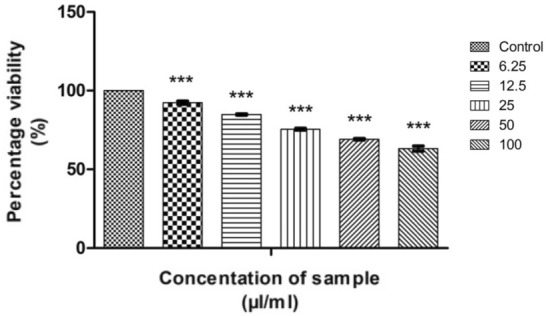
Graphical representation depicting the cytotoxic effect of CMT on A549 cell lines by MTT assay. Data was shown as the mean ± standard deviation (experiments = 3), ***p < 0.001 compared to untreated cells.

**Figure 14:**
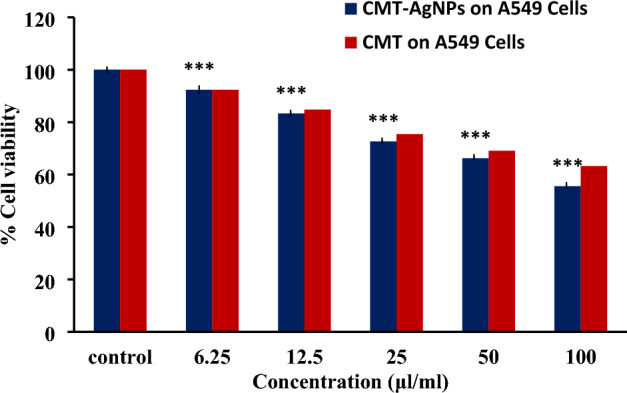
Comparison of anticancer activity of CMT and CMT-AgNPs against A549 cell lines (human lung cancer cells). Data was shown as the mean ± standard deviation (experiments = 3), ***p < 0.001 compared to untreated cells.

The measure of cell survival or healthy cells is represented by cell viability value^76^. The MTT assay results of CMT-AgNPs and CMT on A549 cell lines are shown in Figs. 12 and 13 respectively. It is the graphical representation showing the % cell viability upon the addition of increasing concentrations of CMT-AgNPs. From both graphs, we inferred that the cytotoxicity on A549 cells is increasing with increasing concentrations. Figure 14 is a comparison between the anticancer activity of camptothecin and CMT-AgNPs. It is clearly showing that CMT-AgNPs is having higher activity than CMT. Moreover, the increase in the activity of nanoparticles is faster than that of CMT. The images of A549 cells morphology after the treatment of different concentrations of samples (after 24 h of incubation) are given as supplementary data (S1, S2). S1 and S2 are the microscope images of A549 cells treated with CMT-AgNPs and CMT respectively. The images of control cells were looking clear and tightly packed. But detectable changes were observed in the morphology of treated cells. More shrinking and rounding were seen while increasing the concentrations of CMT-AgNPs. More collapsed morphology is shown in CMT-AgNPs treated A549 cells than CMT treated ones. The LC50 value is calculated from the results using ED50 PLUS V1.0 Software. LC50 value for CMT and CMT-AgNPs against A549 cell lines were calculated as 135.8339 µl/ml and 107.1867 µl/ml respectively.

**Figure 15:**
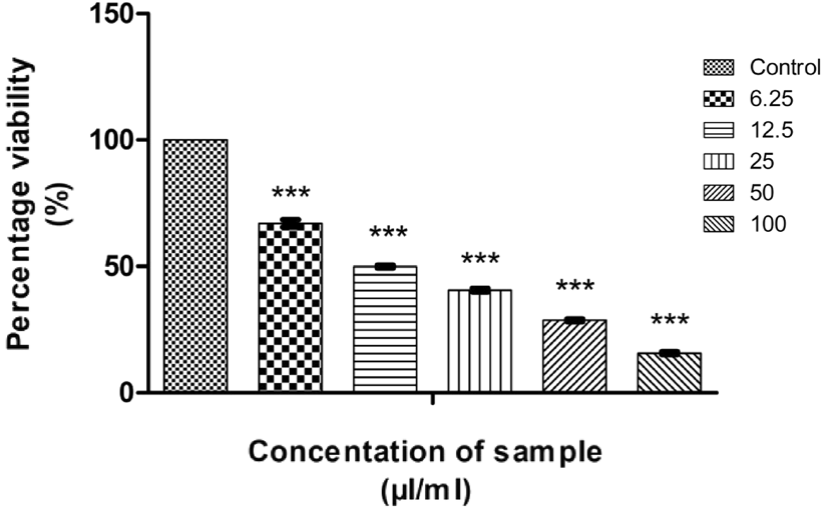
Graphical representation depicting the cytotoxic effect of Doxorubicin on A549 cell lines by MTT assay. Data was shown as the mean ± standard deviation (experiments = 3), ***p < 0.001 compared to untreated cells.

We have taken doxorubicin as a positive control to compare CMT-AgNPs. It is considered one of the strongest chemotherapy drugs^71,72^. The MTT assay of doxorubicin is shown in Fig. 15. Morphology changes of the A549 cell lines are shown in supplementary data S3. Also, the LC50 value is calculated to be 12.47858 µg/ml. When comparing with CMT-AgNPs, doxorubicin shows higher anticancer activity. But doxorubicin is a synthetic drug and our sample is a natural drug functionalised nanoparticle^77^. While taking the unique properties of nanoparticles into account, we cannot directly compare a synthetic drug with nanoparticles. However, our sample is showing higher anticancer activity than a known natural anticancer drug, camptothecin. Hence more studies are needed to confirm the potential of CMT-AgNPs as an anticancer drug.

#### Statistical analysis

The experiments were done in triplicates and results are shown as Mean ± standard deviation. One-way ANOVA and Dunnett's test were conducted to analyse data. A value of P < 0.001 is considered as a statistically significant data. Hence all the results are statistically relevant.

##### Detection of ROS generation

**Figure 16:**
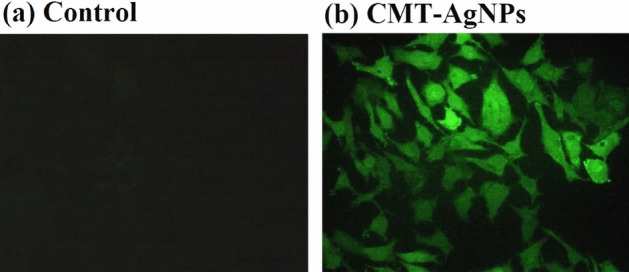
Images of ROS damage of A549 cells (**a**) untreated control, (**b**) treated with CMT-AgNPs.

**Figure 17:**
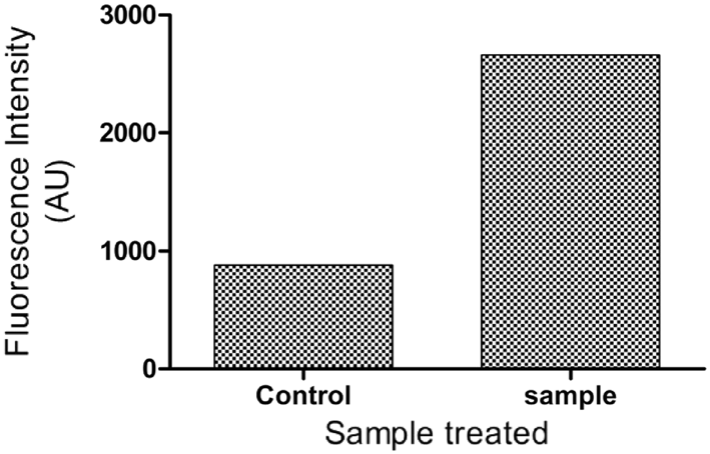
Fluorescence intensity of A549 cells from DCFDA staining.

Intracellular reactive oxygen species (ROS) are produced primarily by mitochondria and has a key role in cancer therapy^78^. The lower levels of ROS are acceptable in cells, but the excessive quantity will result in cell death. Hence, induction of ROS is a favourable strategy to develop cancer therapy. i.e., a cancer drug which produces higher levels of ROS will cause oxidative stress to kill cancer cells^79–81^. Hence, it is also important to detect the ROS generation and apoptosis to further confirm the activity. For this LC50 concentration of CMT-AgNPs was treated to A549 cells and DCFDA staining was used to find the cell damage through ROS production^80^. The ROS generation and corresponding morphological changes in A549 cells were recorded using a fluorescence microscopy. Figures 16 and 17 are the ROS damage and the fluorescence intensity of the cell lines treated with CMT-AgNPs. Cells treated with nanoparticles shows a florescence intensity of 2662.30 AU, which suggests the higher ROS generation level when comparing with untreated control cells (880.17). Also, the bright green florescence of CMT-AgNPs treated cells in Fig. 16 proposing a high level of intracellular ROS generation after 24 h of incubation. It is also clear that the control cells significantly reduced the ROS generation. The overall study suggests an exquisite ROS generation capacity of CMT-AgNPs against human lung cancer cell lines at their LC50 dose.

##### Flow cytometry to measure apoptosis

**Figure 18:**
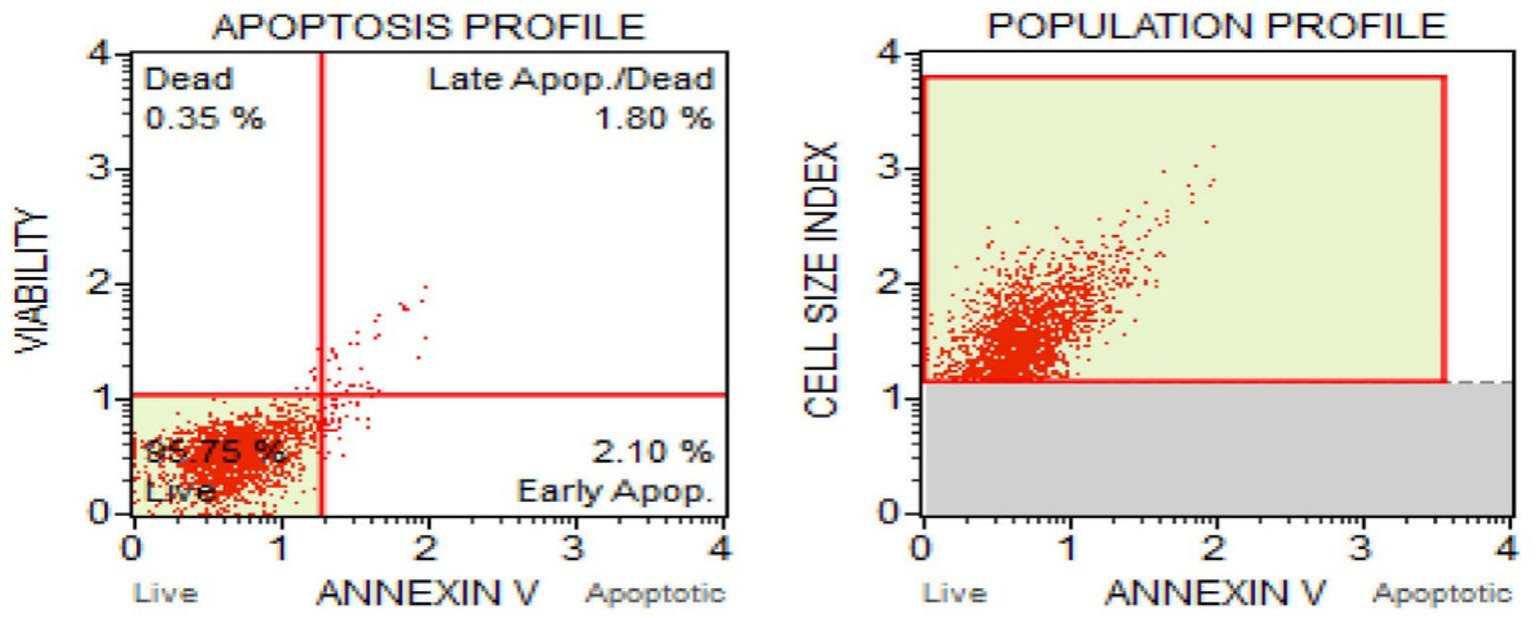
Apoptosis and population profiles of untreated control cells.

**Figure 19:**
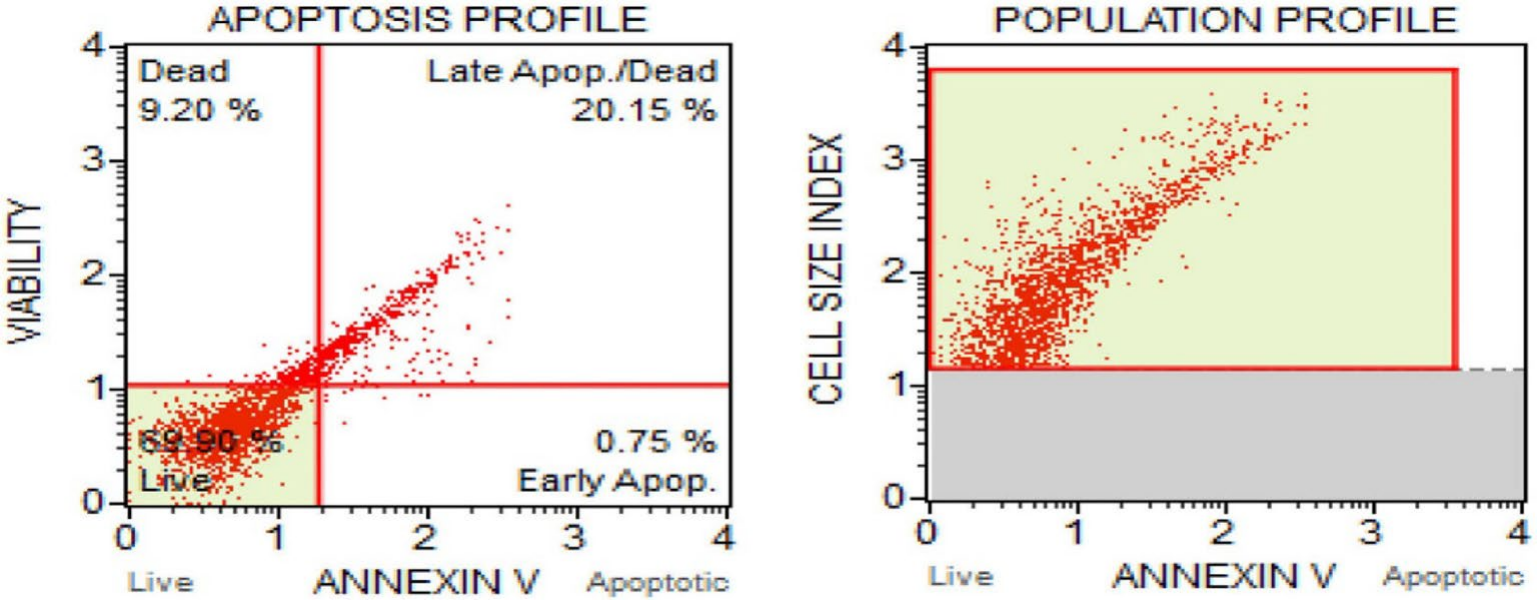
Apoptosis and population profiles of A549 cells treated with CMT-AgNPs.

Apoptosis is a type of natural cell death through which the body clear out the abnormal cells^82^. It is an extremely effective elemental suicide pathway. Hence a drug that trigger apoptosis might be acceptable for cancer therapy^83,84^. Flow cytometry is an effective method for evaluating apoptosis^85^. This provides a methodical procedure to identify cells separately instead of analysing a mixed population. The FITC Annexin V/Dead Cell Apoptosis Kit with FITC annexin V and PI (propidium iodide) for flow cytometry provides a quick and well-suited assay for apoptosis^23,43^. PI is a red-fluorescent dye which can stain dead cells through binding with nucleic acids and impermeable to apoptotic cells and live cells. Figures 18 and 19 represents the population profile and apoptosis profile of untreated and treated cells (with CMT-AgNPs) respectively. Cell distribution in A549 cells treated with CMT-AgNPs and control cells were shown in supplementary data S4. From the results, it can be observed that when treated with LC50 value, the percentage of live cells decreased to 69% from 95% compared with untreated control cells. Treatment with CMT-AgNPs increased the percentage of apoptotic cells from 3 to 20% suggesting induction of apoptosis upon compound treatment.

#### In vitro MTT assay on cervical and colon cancer cells

**Figure 20:**
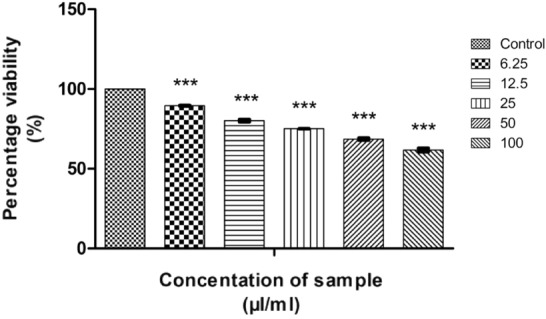
Graphical representation depicting the cytotoxic effect of CMT-AgNPs on HT29 cell lines by MTT assay. Data was shown as the mean ± standard deviation (experiments = 3), ***p < 0.001 compared to untreated cells.

**Figure 21:**
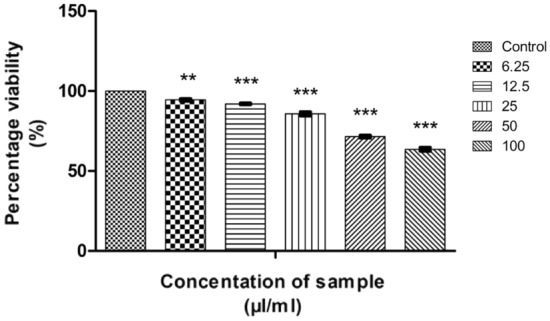
Graphical representation depicting the cytotoxic effect of CMT-AgNPs on HeLa cell lines by MTT assay. Data was shown as the mean ± standard deviation (experiments = 3), ***p < 0.001 compared to untreated cells, **p < 0.01 compared to untreated cells.

In vitro MTT assays were conducted on HT29 and HeLa cell lines to confirm the anticancer potential of CMT-AgNPs. Figures 20 and 21 are the graphical representations illustrating the cytotoxic effect of CMT-AgNPs on HT29 and HeLa cell lines by MTT. Similar to A549, cytotoxicity of CMT-AgNPs on HT29 and HeLa cell lines are also increasing in dose dependent manner. But the calculated LC50 is higher than A549 cell lines. The LC50 value for HT29 cell lines is 132.5436 µl/ml and for HeLa cell lines is 135.1747 µl/ml. Morphology changes of the colon and cervical cancer cell lines while applying the CMT-AgNPs were given in supplementary data S5 and S6. This confirmed that the CMT-AgNPs has anticancer potential on both HT29 and HeLa cell lines, although it shows more activity towards human lung cancer cells.

**Figure 22:**
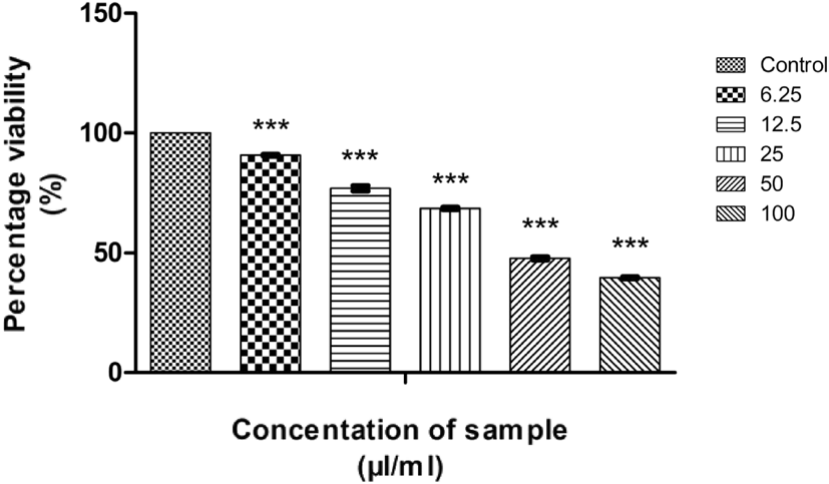
Graphical representation depicting the cytotoxic effect of CMT on HT29 cell lines by MTT assay. Data was shown as the mean ± standard deviation (experiments = 3), ***p < 0.001 compared to untreated cells.

**Figure 23:**
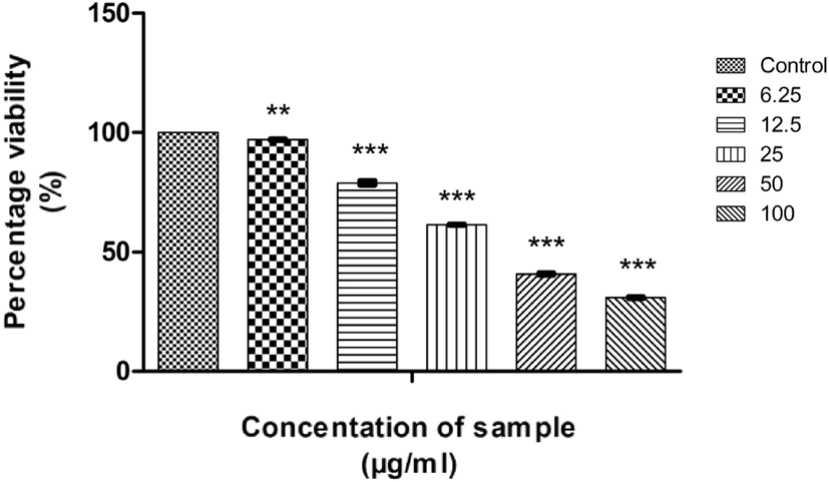
Graphical representation depicting the cytotoxic effect of CMT on HeLa cell lines by MTT assay. Data was shown as the mean ± standard deviation (experiments = 3), ***p < 0.001, **p < 0.01 compared to untreated cells.

To compare the anticancer activity, in vitro MTT assays were conducted with camptothecin against HT29 and HeLa cell lines and their graphical representations are shown in Figs. 22 and 23. The morphology changes of the colon and cervical cancer cell lines while applying CMT were given in supplementary data S7 and S8. A comparison graph of the anticancer activity of camptothecin and CMT-AgNPs against both the cells were given in supplementary data S9 and S10. The calculated LC50 value of CMT for HT29 and HeLa cell lines are 46.42733 µl/ml and 39.6765 µl/ml respectively**.** These values are much lower than those obtained for CMT functionalised nanoparticles. Our experiments established that CMT has higher anticancer activity towards HeLa and HT29 cell lines when compared with CMT-AgNPs. Conversely, the latter shows higher activity towards lung cancer cells than CMT. Hence, all these results confirm that CMT-AgNPs have higher anticancer potential towards lung cancer cells.

#### In vitro MTT assay on L929 cell lines

**Figure 24:**
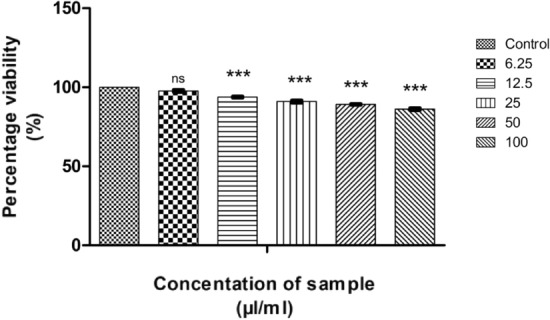
Graphical representation depicting the cytotoxic effect of CMT-AgNPs on L929 cell lines by MTT assay. Data was shown as the mean ± standard deviation (experiments = 3), ***p < 0.001 compared to untreated cells, ns- not significant.

**Figure 25:**
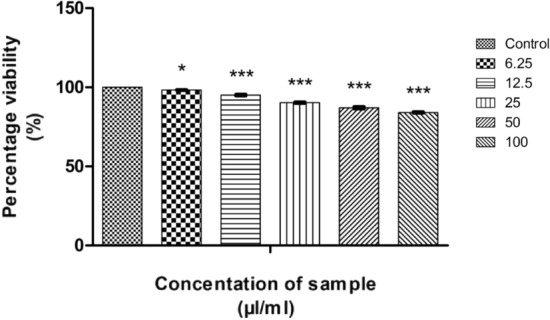
Graphical representation depicting the cytotoxic effect of CMT on L929 cell lines by MTT assay. Data was shown as the mean ± standard deviation (experiments = 3), ***p < 0.001, *p < 0.1 compared to untreated cells.

**Figure 26:**
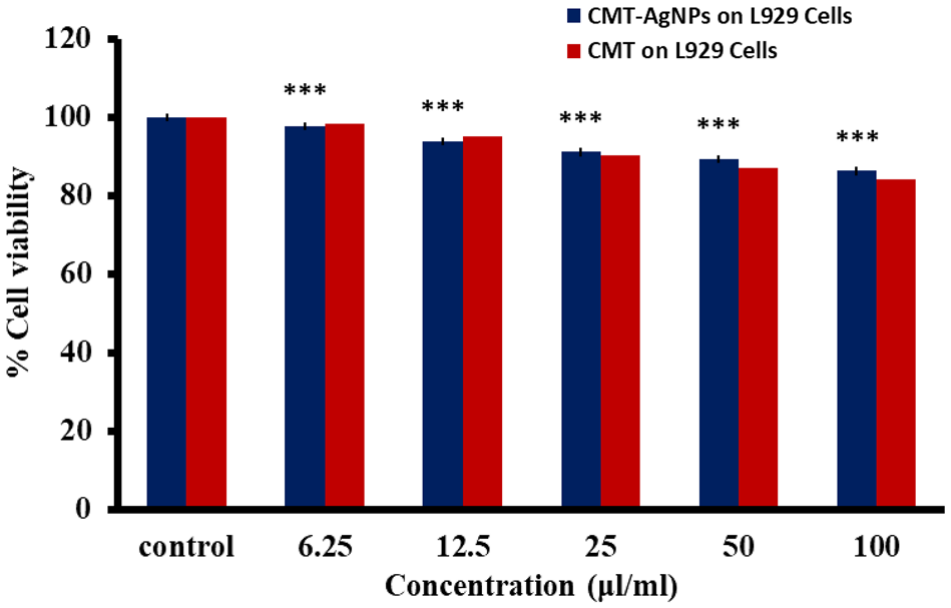
Comparison of cytotoxicity of CMT and CMT-AgNPs against L929 cell lines. Data was shown as the mean ± standard deviation (experiments = 3), ***p < 0.001 compared to untreated cells.

According to ISO 10993-5:2009, the cytotoxicity analysis of CMT-AgNPs and CMT were conducted on L929 cell lines^86–88^. Figures 24 and 25 are the graphical representations illustrating the cytotoxic effect of CMT-AgNPs and CMT respectively. Even though the cytotoxicity of the prepared nanoparticles is increasing in a concentration dependent manner, the rate of increase is very low. The exposure of L929 cells to 6.25 µl/ml of CMT-AgNPs decreased the cell viability to 97.68%. However, when the concentration increased to 100 µl/ml, the viability is only decreased to 86.20%. Similarly, treating the cells with 100 µl/ml of CMT, the cell viability decreased to 84%. Figure 26 is a comparison between the cytotoxicity of camptothecin and CMT-AgNPs. This clearly shows the higher toxicity of CMT towards L929 cell lines when compared with CMT-AgNPs. Morphology changes of the L929 cell lines while applying the CMT-AgNPs and CMT were given in supplementary data S11 and S12, respectively. The images of control cells were looking clear and tightly packed. But detectable changes were observed in the morphology of treated cells. The changes were more visible in CMT treated L929 cells. LC50 value of CMT-AgNPs and CMT are calculated to be 437.93027 µl/ml and 336.68574 µl/ml respectively**.** This values also verify the lower cytotoxicity of CMT-AgNPs when compared with CMT. Hence the results once again substantiate the potential of CMT-AgNPs in biomedical applications^89–92^.

## Conclusion

We have investigated the interaction strategy of CMT-AgNPs with ctDNA through various spectroscopic and calorimetric techniques. The CMT-AgNPs were successfully synthesised using camptothecin as both reducing and capping agent through the one-pot synthesis method. The prepared CMT-AgNPs are stable and have an average particle size of 10 ± 2 nm. The intensity variations in the UV-absorbance spectrum of ctDNA with varying concentrations of CMT-AgNPs and vice-versa indicate that CMT-AgNPs bind to ctDNA. The competitive dye displacement studies with MetG (major groove binder) and Hst (minor groove binder) dyes confirmed the major groove binding mode of CMT-AgNPs to the ctDNA with a high binding energy constant in the order of 10^4^ M^−1^. The viscosity analysis of ctDNA with different concentrations of CMT-AgNPs further verifies the groove binding chances inferred from the fluorescent dye displacement studies. The thermodynamic parameters extracted from the isothermal titration calorimetry favoured a negative enthalpy of binding with an average value of −31.94 kJ/mol and hence the reaction is exothermic and spontaneous. Also, CMT-AgNPs–ctDNA interaction is enthalpy driven which again confirms the possibility of major groove binding. The binding constants obtained from the UV absorption experiments, fluorescence dye displacement studies and ITC maintained consistency and the value is in the order of 10^4^ Mol^−1^. CD spectra were analysed to find the stability of ctDNA when complexed with CMT-AgNPs. Minimal variations in the CD spectra confirm the insignificant conformational changes of ctDNA and hence the unaltered functions of ctDNA while interacting with CMT-AgNPs. We have uncovered the therapeutic effects of CMT-AgNPs on cancer treatment. An exhaustive in vitro MTT assay on A549 cells, HeLa cells and HT29 cells revealed the good anticancer activity of CMT-AgNPs. From the results it is confirmed that the CMT-AgNPs has higher activity towards human lung cancer cell lines. To further confirm the results, flow cytometry and ROS production is studied. All the analyses propose the higher anticancer activity of CMT-AgNPs towards A549 cell line. An exhaustive in vitro MTT assay of CMT-AgNPs against L929 cell lines showed a very low cytotoxicity which indicating the possibility of using CMT-AgNPs on medical devices. Overall, we believe that camptothecin functionalised silver nanoparticles is a potential prospect for pharmaceutical applications.

## Supplementary Information

Supplementary Information.

## Data Availability

All the images and tables given in the article are obtained based on experimental data. None of the images were reproduced from other sources. The datasets used and/or analysed during the current study available from the corresponding author on request.
