# Supplementary material for: Exploring the cytotoxicity on human lung cancer cells and DNA binding stratagem of camptothecin functionalised silver nanoparticles through multi-spectroscopic, and calorimetric approach
Source: Sci Rep. 2023 Jun 3;13:9045. doi: 10.1038/s41598-023-34997-w (PMC10239481; doi:10.1038/s41598-023-34997-w)
Supplement: Supplementary file 1 — Supplementary Information. [file 41598_2023_34997_MOESM1_ESM.docx]

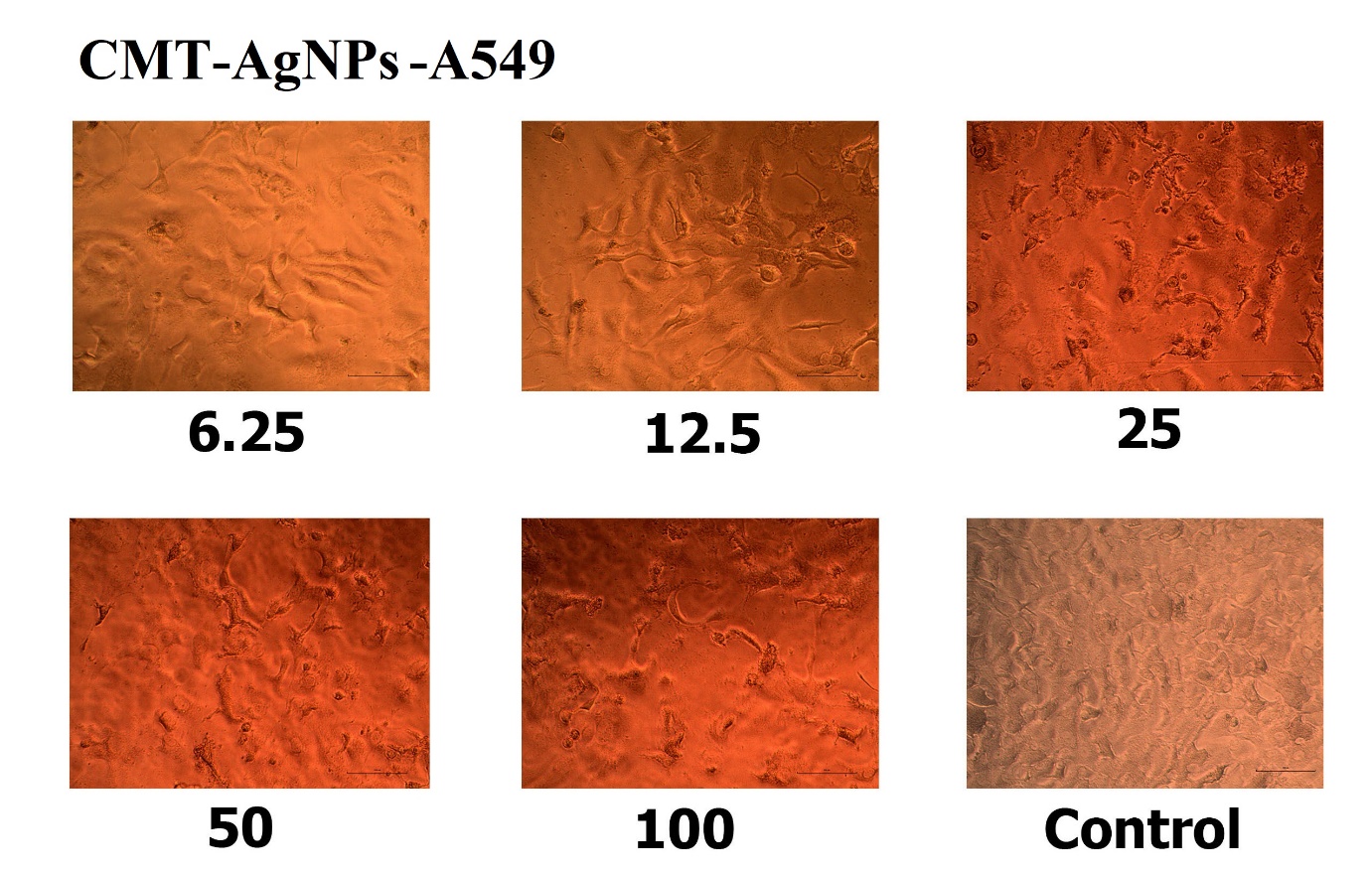


**S1: The observed cell morphology of A549 cell lines treated with CMT-AgNPs through phase contrast microscopy**


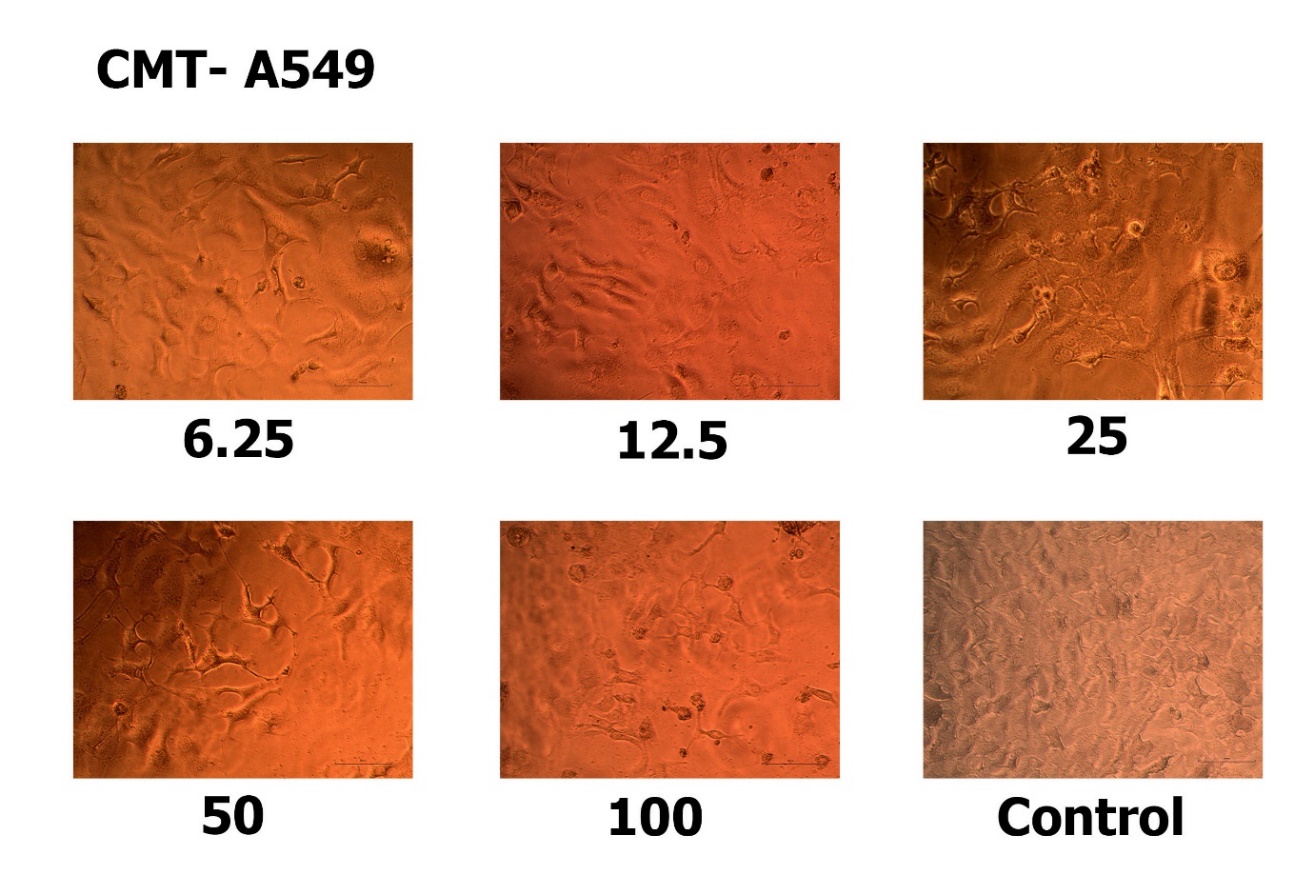


**S2: The observed cell morphology of A549 cell lines treated with CMT through phase contrast microscopy**


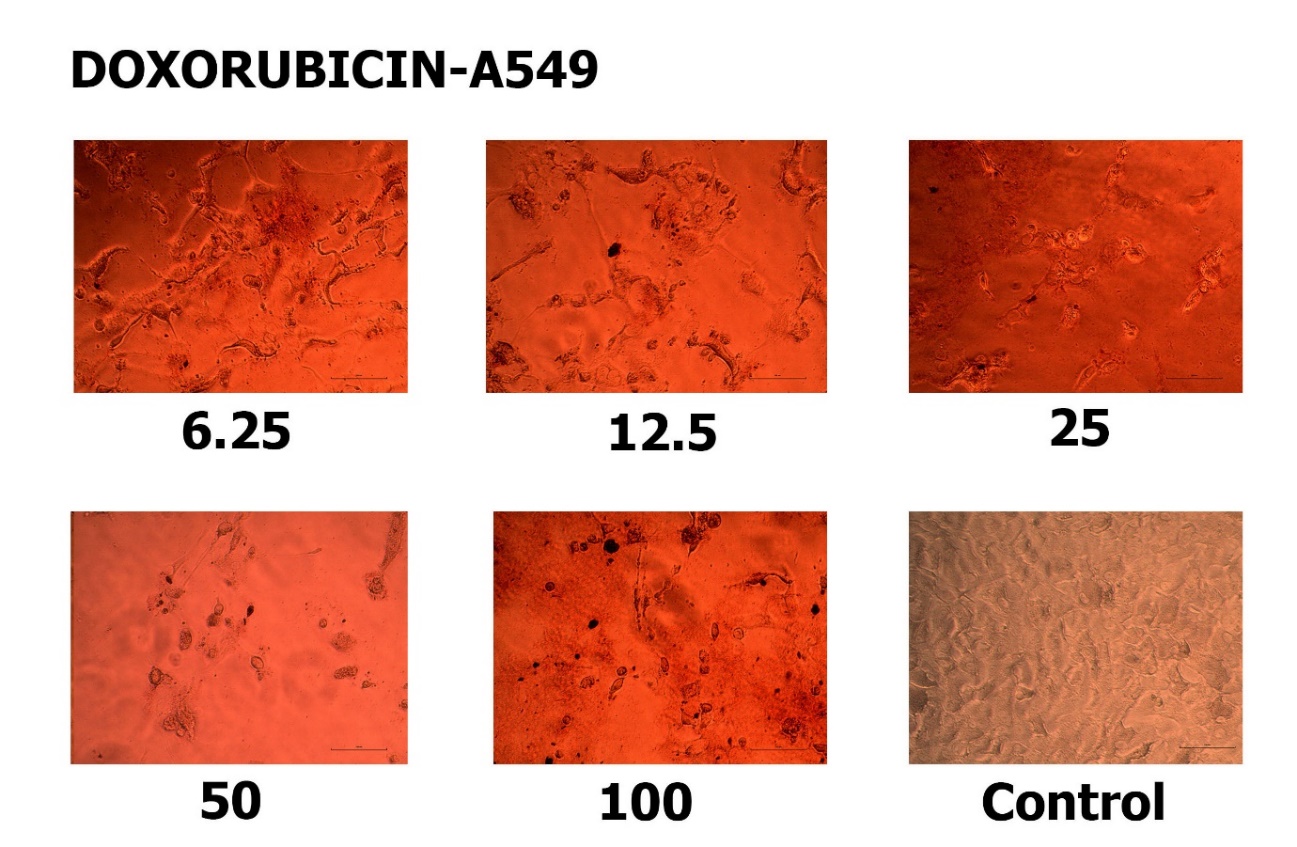


**S3: The observed cell morphology of A549 cell lines treated with doxorubicin through phase contrast microscopy**

**
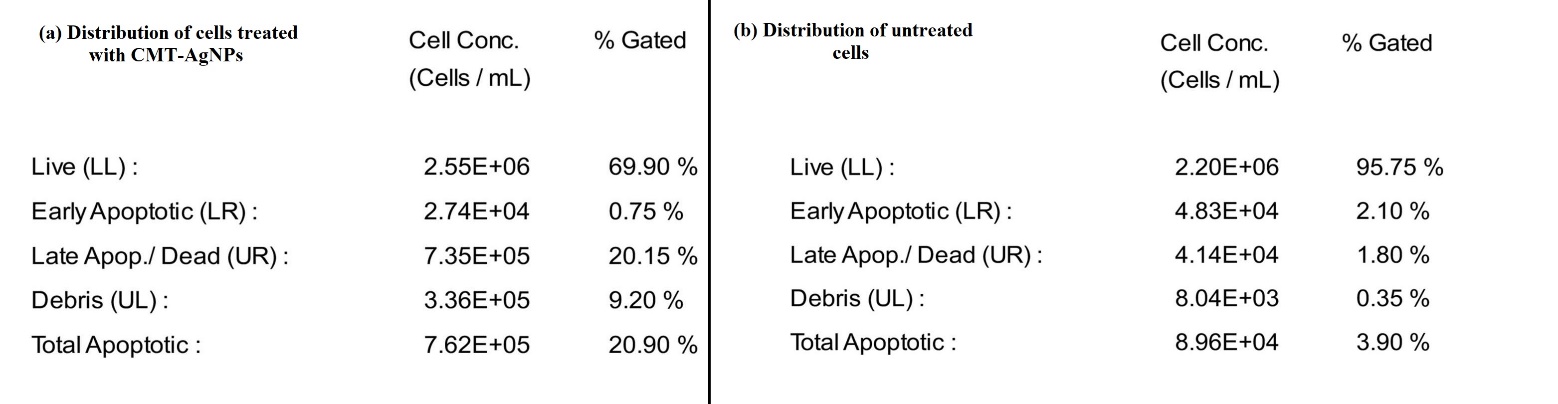
**

**S4: Cell distribution in A549 cells treated with (a) CMT-AgNPs and (b) untreated control cells**

**
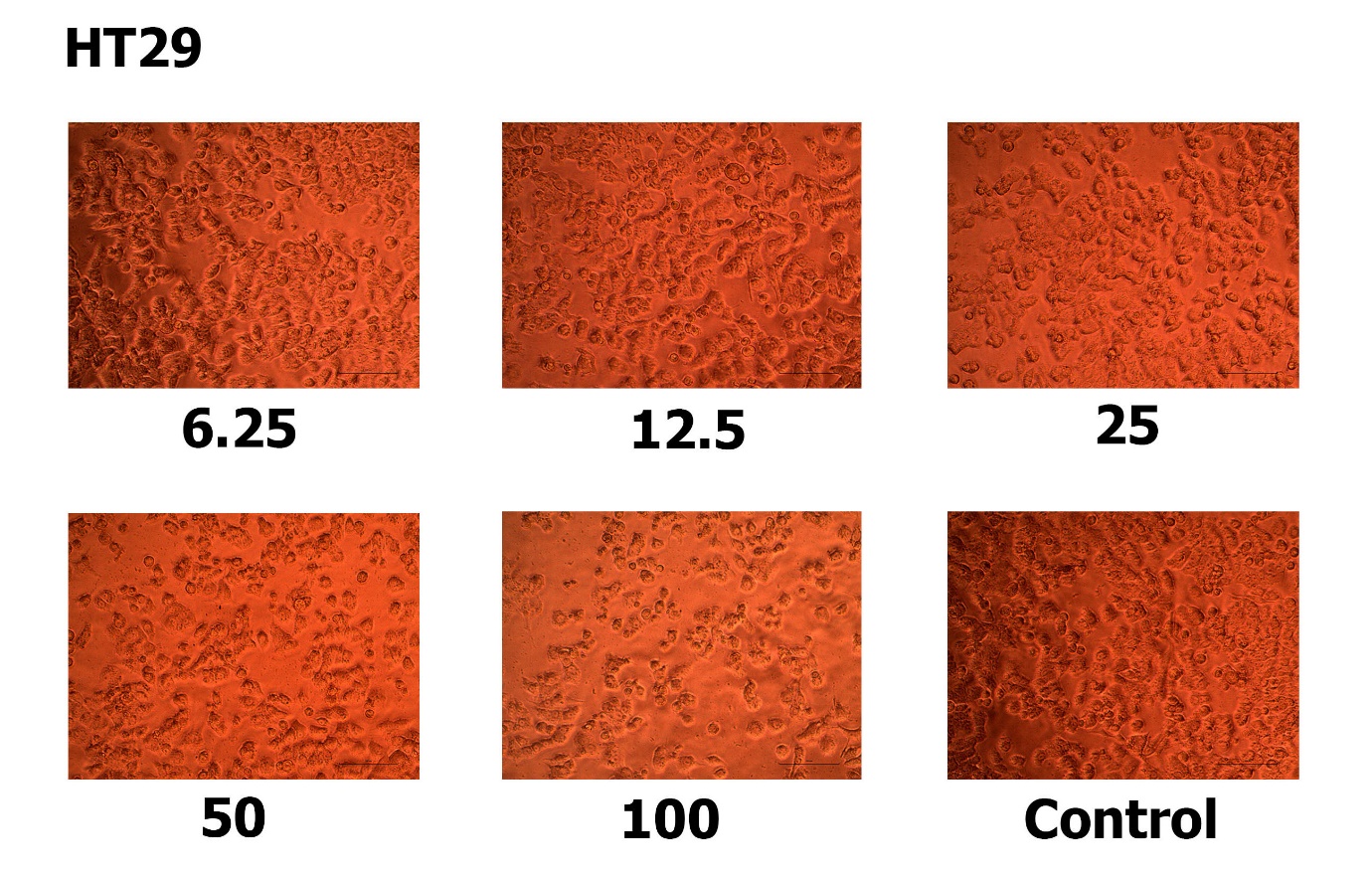
**

**S5: The observed cell morphology of HT29 cell lines treated with CMT-AgNPs through phase contrast microscopy**

**
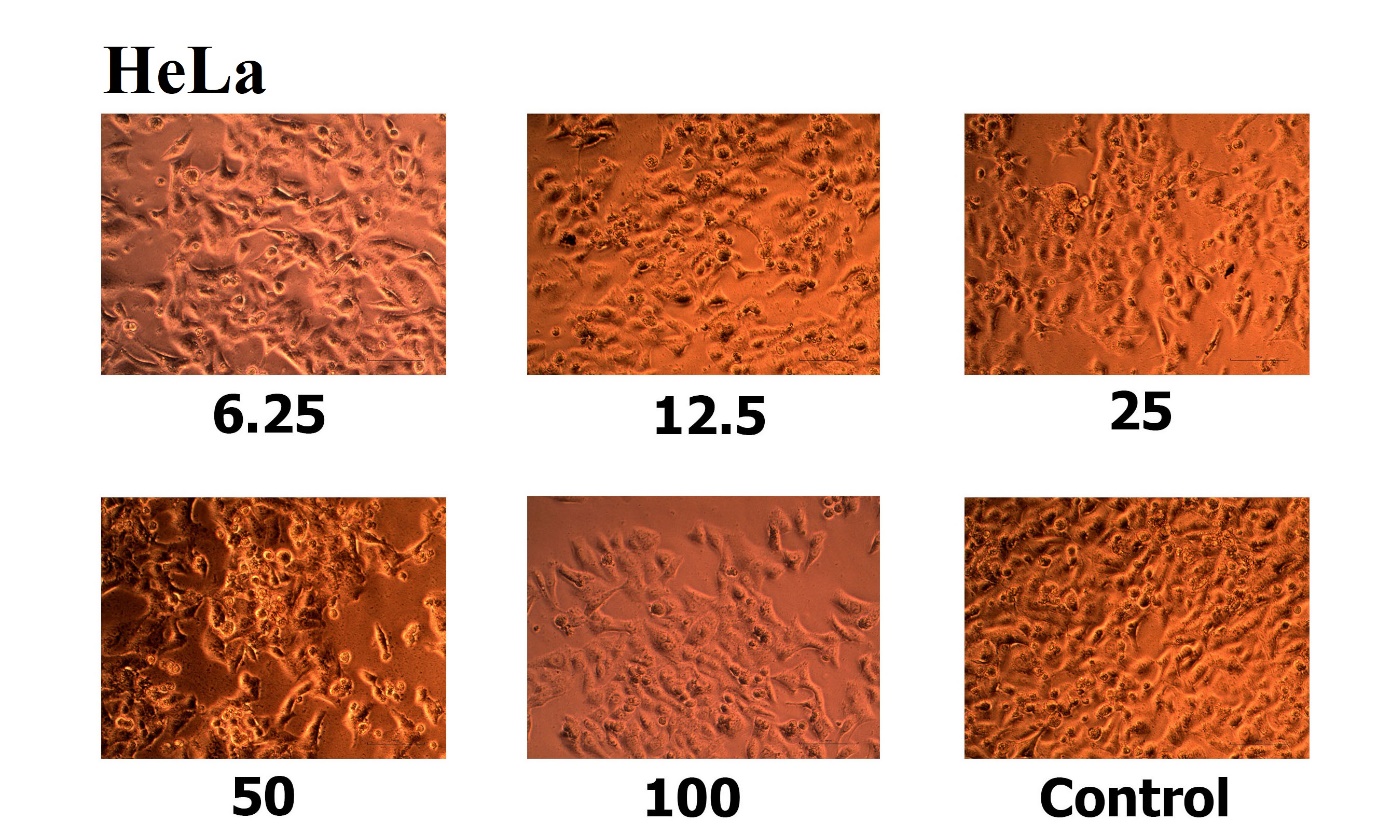
**

**S6: The observed cell morphology of HeLa cell lines treated with CMT-AgNPs through phase contrast microscopy**

**
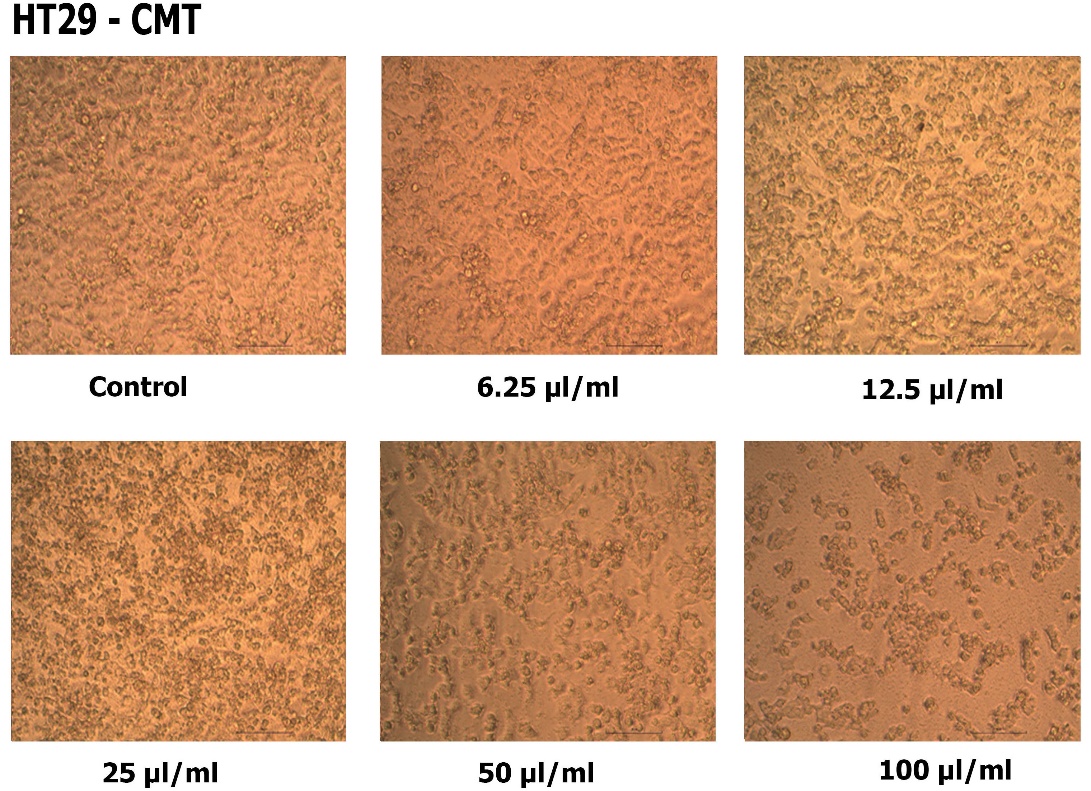
**

**S7: The observed cell morphology of HT29 cell lines treated with CMT through phase contrast microscopy**

**
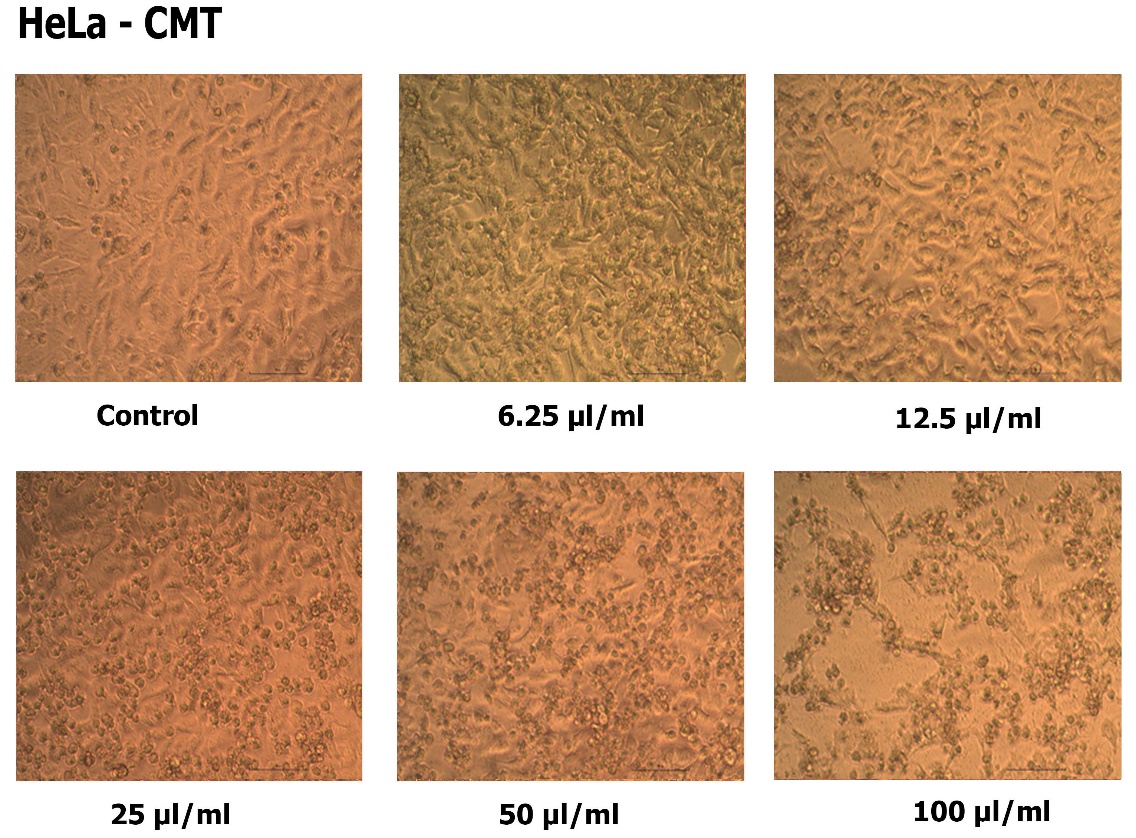
**

**S8: The observed cell morphology of HeLa cell lines treated with CMT through phase contrast microscopy**

**
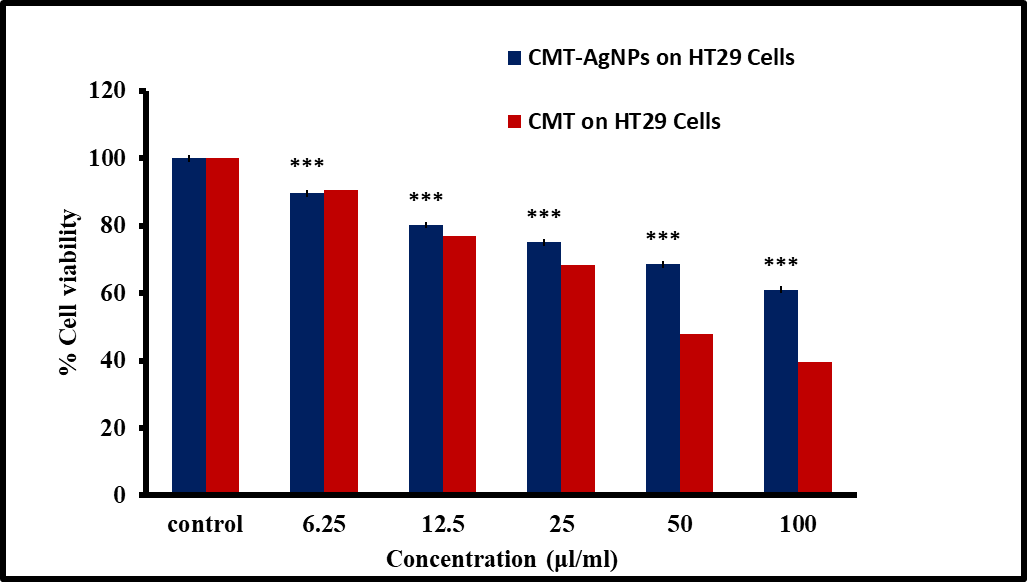
**

**S9: Comparison of anticancer activity of CMT and CMT-AgNPs against HT29 cell lines. Data was shown as the mean+/- standard deviation (experiments=3), ***p< 0.001 compared to untreated cells.**

**
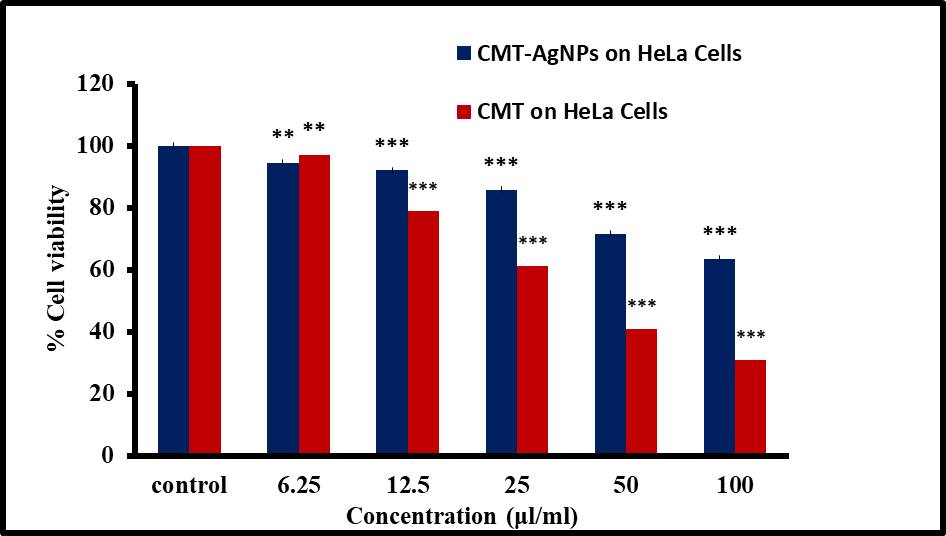
**

**S10: Comparison of anticancer activity of CMT and CMT-AgNPs against HeLa cell lines. Data was shown as the mean+/- standard deviation (experiments=3), ***p< 0.001, **p<0.01 compared to untreated cells.**

**
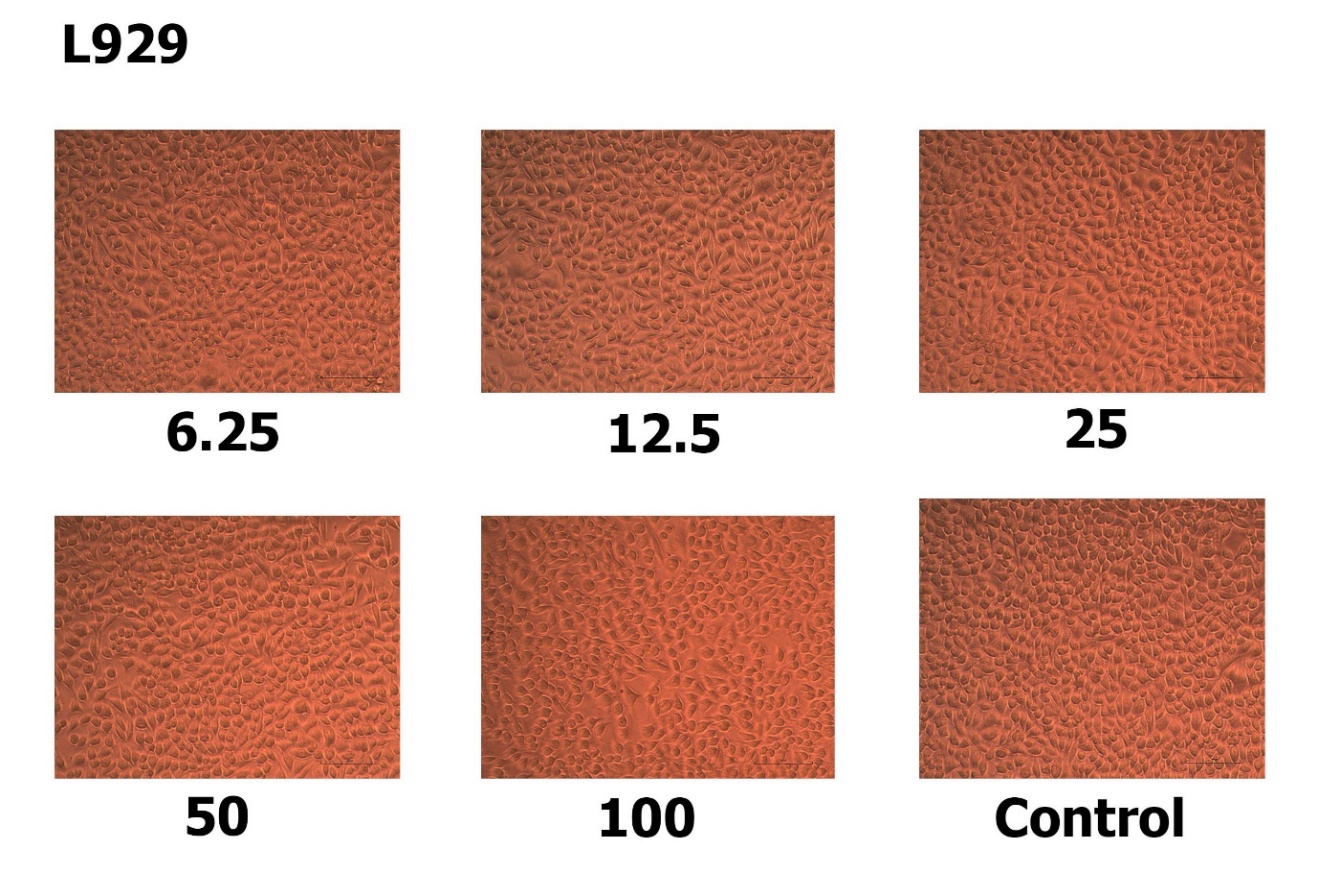
**

**S11: The observed cell morphology of L929 cell lines treated with CMT-AgNPs through phase contrast microscopy**

**
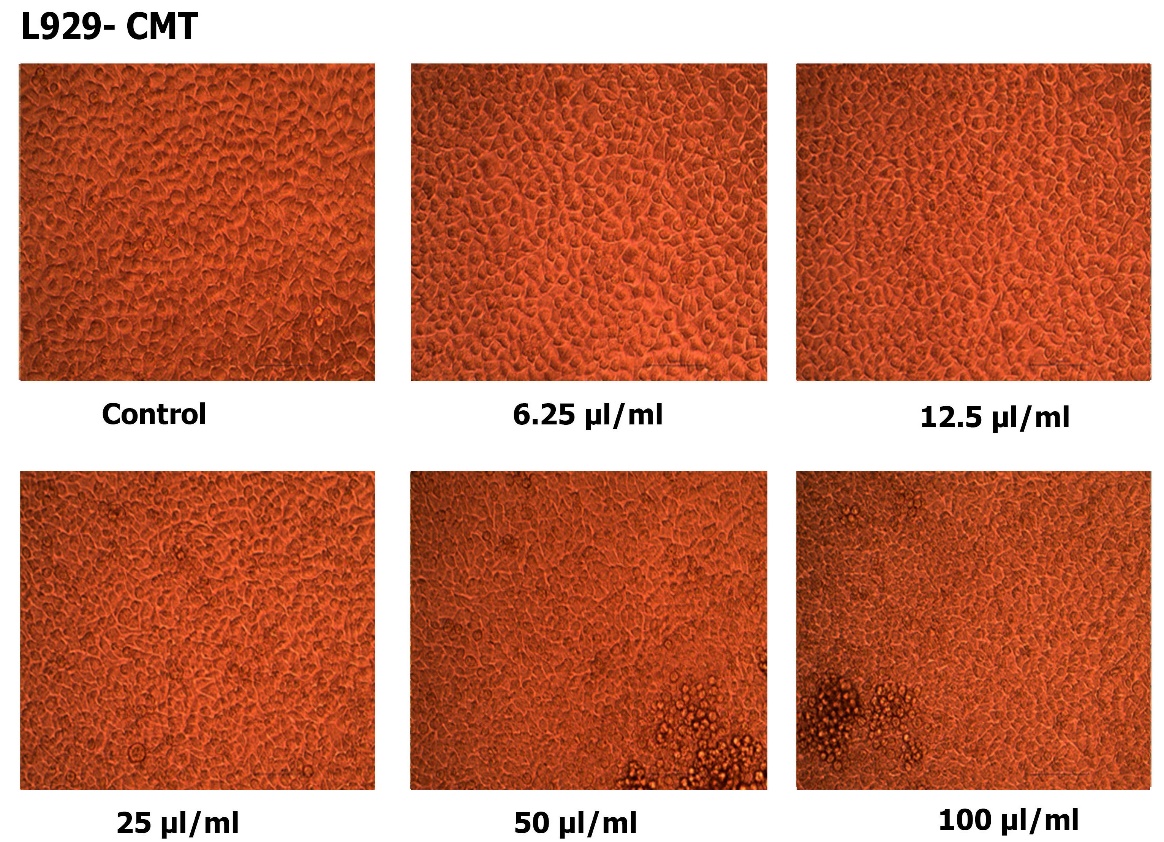
**

**S12: The observed cell morphology of L929 cell lines treated with CMT through phase contrast microscopy**
